# Associations Between Weight Status and Mental Well‐Being in Childhood and Adolescence: A Systematic Review of Longitudinal Studies

**DOI:** 10.1111/obr.70169

**Published:** 2026-06-09

**Authors:** Stine Schramm, Iben Rask Heuck, Nina Rica Wium Geiker, Cathrine Lawaetz Wimmelmann

**Affiliations:** ^1^ Centre for Childhood Health Copenhagen Denmark

**Keywords:** childhood, mental well‐being, obesity, overweight, quality of life, self‐esteem, weight status

## Abstract

**Introduction:**

Obesity and poor mental well‐being in childhood and adolescence are growing public health concerns with potential implications for a broad range of life outcomes. Understanding the longitudinal and potentially bidirectional relationship between weight status and mental well‐being is crucial for developing effective interventions to support children and adolescents.

**Methods:**

This systematic review synthesizes evidence on the bidirectional relationship between weight status and indicators of mental well‐being over time. Peer‐reviewed studies published up to January 10, 2025 were identified through searches in PubMed, PsycINFO, Scopus, and Web of Science. Eligible studies included longitudinal designs assessing weight status and mental well‐being in populations up to age 18 years. The Joanna Briggs Instrument assessed the risk of bias, and the findings were narratively summarized.

**Results:**

The review included 33 longitudinal studies conducted between 1994 and 2022. We found that higher childhood weight was associated with subsequent low mental well‐being, and an indication that poor mental well‐being was associated with subsequent higher weight. However, there was a scarcity of longitudinal studies exploring the influence of mental well‐being on subsequent weight status. Large heterogeneity in design and methods was found across the included studies.

**Conclusion:**

Findings indicated a bidirectional association between weight status and mental well‐being, with stronger evidence supporting the impact of weight status on mental well‐being than vice versa. Future research should prioritize longitudinal designs to better understand the influence of mental well‐being on later weight development and explore underlying mechanisms.

**Abbreviations**
BMI: body mass index
CBCL: child behavior checklist
CDC: Center for Disease and Control and Prevention
CHQ‐PF50: Child Health Questionnaire—Parent Form 50
CRS: The Conner's Rating Scale
DEXA‐scan: dual‐energy x‐ray absorptiometry (DEXA)‐scan
HBSC: The Health Behavior in School‐Aged Children Study
HRQoL: health‐related quality of life
IOTF: International Obesity Task Force
PANAS‐C: The Positive Affect and Negative Affect Schedule—Child Form
PedsQL: pediatric quality of life inventory
PSS: Perceived Stress Scale
QoL: quality of life
RSE: Rosenberg Self‐Esteem
SDQ: Strengths and Difficulties Questionnaire
SPPA/SPPC: The Harter Self‐Perception Profile for Adolescents/Children
SRS: Social Rating Scale—adapted from the Social Skill Rating System
SSRS: Social Skills Rating System
TBAQ: Toddler Behavior Assessment Questionnaire
WHO: World Health Organization
WHtR: waist‐to‐height ratio

## 1 Introduction

High body mass index (BMI) and poor mental health in childhood and adolescence are both growing public health concerns [1, 2] that may have serious short and long‐term consequences [3, 4]. Although the prevalence of childhood overweight and obesity has steadily increased in most Western countries over the last three decades, a concurrent increase in the prevalence of poor mental well‐being among children and adolescents has been observed, starting around the year 2010 [5, 6, 7]. These trends are accompanied by a marked increase in the prevalence of psychiatric diagnoses among children and adolescents [2] with 8% of children and 15% of adolescents globally receiving a psychiatric diagnosis [8]. Moreover, research has consistently found evidence of a link between obesity and poor mental health and well‐being in childhood and adolescence [9]. For instance, a growing body of longitudinal studies has reported that children and adolescents with obesity have an increased risk of developing depression compared to children and adolescents with a normal weight [10, 11]. In a meta‐analysis of 10 longitudinal studies, Sutaria et al. [10] found that children with obesity had 51% higher odds of developing depression over time compared to children without obesity.

The influence of obesity on later mental health likely reflects mechanisms such as bullying victimization [12], societal stigma [13], low self‐esteem [14, 15], and the physical health challenges associated with excess weight [16]. Conversely, it has been suggested that poor mental health and well‐being are associated with behaviors and biological processes involved in regulating the energy‐balance, such as endocrine disturbances including stress reactivity [17], disordered eating patterns [18], lack of physical activity [19], and poor sleep quality [20], consequently increasing the risk of overweight and obesity [21, 22]. Thus, it is believed that there is a bidirectional relationship between obesity and poor mental health, where psychological distress promotes unhealthy weight gain, and a high weight status further worsens mental health issues, making it increasingly difficult for affected children and adolescents to break out of this detrimental cycle.

Mental well‐being is typically defined according to WHO's concept of mental health; a state of mental well‐being that enables people to cope with the stresses of life, realize their abilities, learn and work well, and contribute to their community. Here, mental health conditions include mental disorders and psychosocial disabilities, as well as other mental states. However, an important distinction concerns the difference between mental health and mental well‐being. Mental well‐being is a non‐clinical construct of mental health and is often defined in terms of several indicators including self‐esteem, self‐efficacy, health‐related quality of life (HRQoL), satisfaction with life, and more functional aspects such as resilient coping with life and functional behavior [23]. Thus, psychiatric symptoms and disorders including depression and anxiety are not encompassed by this construct of “mental well‐being”; however, consistent poor mental well‐being increases the risk of developing such clinical states [24].

Several reviews have been published on the association between weight status and clinical states such as depression and anxiety [10, 11, 25]. In a recent study including children with overweight and obesity, it was found that better mental health and psychosocial well‐being at age 11 years was associated with decreased BMI over time compared with children with lower mental health [26]. Moreover, a recent study has suggested that self‐concept and the degree of behavioral difficulties among 9‐ and 14‐year‐old children mediate the association between childhood adversities and BMI at age 18 years [27]. Recent research has increasingly investigated the direct association between indicators of mental well‐being and weight status, but the overall picture of this research remains unclear. Therefore, this review aimed to systematically review existing longitudinal studies investigating the potentially bidirectional association between poor mental well‐being and weight status among children and adolescents. Focusing on the non‐clinical construct of mental health, this study focused on mental well‐being measures, including self‐esteem, socioemotional skills, and HRQoL. A more comprehensive understanding of the potential bidirectional relation between mental well‐being and weight status over time may contribute with important knowledge for prevention purposes both in childhood and youth as well as throughout life.

## 2 Materials and Methods

### 2.1 Data source and Literature Search

A systematic literature search was conducted in the following four databases: PubMed, PsycINFO, SCOPUS, and Web of Science. In all four databases, the search strategy used Boolean terms such as “AND” and “OR” and truncations in between the search terms. In PubMed, the search string included Medical Subject Headings (MESH‐terms) in addition to title and abstract searches. In PsycINFO, the search string included Thesaurus terms in addition to abstract searches. The search string has been set up in accordance with the main research question and revised to fit each of the included bibliographic databases. Several literature search specialists and information specialists were consulted to help refine the search string and tailor it to the individual databases. Only English language and peer‐reviewed publications were included. All eligible studies dating to the time of the search were included. The literature search was done on November 23, 2023 and rerun on January 10, 2025. Identified papers were exported to the systematic review processing tool Covidence.

### 2.2 Study Selection Process

The study selection process was performed independently and blinded by two authors. First, a screening was conducted exclusively on the title/abstract (by C.L.W. and S.S.). Secondly, a full‐text screening was performed (by C.L.W. and I.R.H.) on all studies passing the title/abstract screening. Any disagreements between the reviewers were resolved through discussion between the two authors screening and, if necessary, involved a third reviewer (S.S.). After full‐text screening, all eligible papers were exported from Covidence to excel for data extraction and quality assessment.

### 2.3 Eligibility Criteria and Definitions

Originally, the review included quantitative observational studies (including cohort and case–control studies) with both a cross‐sectional and a longitudinal design. However, due to an unexpectedly large number of both cross‐sectional and longitudinal studies, it was decided to focus exclusively on longitudinal research. Hence, cross‐sectional studies were excluded from the current review, but a table of the found cross‐sectional studies and a short synthesis of the results are presented in Table S1. Intervention studies were excluded from the review.

We included studies with the main purpose of performing analyses investigating the longitudinal association of mental well‐being with weight status, vice versa, or a bidirectional association.

Weight status included measures of BMI, BMI *z*‐scores, change in BMI, and other anthropometrics, such as waist circumference, body fat percentage, and waist‐to‐height ratio. Both continuous weight status variables and different categorizations of weight status, i.e., underweight, normal/healthy weight, overweight, and obesity, were eligible for inclusion.

We included different indicators or change in indicators of mental well‐being such as self‐efficacy, self‐esteem, socioemotional skills (referring to the ability to regulate thoughts, emotions, and behaviors), and HRQoL, but excluded indicators of clinical conditions, i.e., psychiatric disorders or symptoms of psychiatric disorders, such as depression and anxiety. Furthermore, assessments of mental well‐being specifically related to weight, such as body dissatisfaction, body image, and body perception, were excluded since research has suggested that such parameters are likely mediators of the mental well‐being—weight status relation [28]. There is a challenge in the transient nature of the phenomena loneliness and bullying, where situational experiences of being alone and ‘minor teasing’, may not reflect sustained distress. Additionally, there is a challenge in measuring loneliness, which is often considered more accurately as an expression of unwanted solitude an objective experience—rather than a global, internalized sense of being alone [29]. Based on these issues, loneliness and bullying were not included as constructs of mental well‐being.

Studies with a study population with an age range between 2 and 18 years both at baseline and follow‐up were included. If the age range exceeded 18 years at follow‐up, the study was included if the majority of participants were under 18 years at follow‐up. Hence, studies measuring the exposure in parents or outcomes during adulthood were excluded. However, the measures were allowed to be proxy‐reported, i.e., by a parent or teacher.

The included studies were limited to Western countries as the discourse related to mental well‐being and especially weight status varies considerably across cultures and since the relationship between weight status and mental well‐being has been shown to differ across countries [30]. Additionally, specific subgroups, such as patient groups, or other selected study populations that do not represent the general population, were excluded. All specific inclusion and exclusion criteria are presented in Table 1.

**TABLE 1:** Inclusion and exclusion criteria.

Inclusion	Exclusion
Main purpose (meaning independent analysis with well‐being and weight as exposure and outcome and preferably adjusted for covariates) Children and adolescents (age 2–18 years) Child‐level measures of weight status and mental well‐being: ○ Assessment of weight status including BMI, BMI *z*‐scores, weight categories, weight change, and other anthropometrics, such as body fat percentage, waist circumference, and waist‐to‐height ratio ○ Assessment of mental well‐being such as quality of life, satisfaction with life, self‐efficacy, general well‐being, socioemotional well‐being, self‐esteem, and stress Longitudinal studies Western countries General population	Newborn and adults Parent‐level measures of weight status and mental well‐being Outcomes in adulthood Assessment of psychiatric disorder or prestates of psychiatric disorders such as depression and anxiety Assessment of mental well‐being specifically related to weight such as body dissatisfaction, body image, body perception, etc. Secondary analysis without adjustment or studies with a different focus that, e.g., assess weight status or mental well‐being as mediators Underweight as the focus of weight status Intervention studies and case–control studies Nonwestern countries Specific subgroups such as patient groups

### 2.4 Data Extraction

The data extraction were performed independently and blinded by two reviewers: S. S or C.L.W. and I.R.H. Any disagreements between the reviewers were resolved through discussion and, if necessary, involved a third reviewer: S. S or C.L.W.

The data extraction included basic information; study author and publication year, study details; study population (including number of participants, age (mean and/or range), and sex), follow‐up period, year of baseline, study location, measurement information on weight status and mental well‐being, and information on the association; study characteristics, measurements on weight and mental well‐being, adjustments, results, and summary conclusion.

### 2.5 Synthesis of Results

As neither concrete exposure nor outcomes were predetermined (specific constructs and instruments), we expected comprehensive methodological heterogeneity among the included studies. Therefore, a basic comparison (vote‐counting) of the number of studies reporting an expected, unexpected, or no association finding was conducted. This categorization was based on the following predefined hypotheses extracted from prior literature: (1) High weight status at baseline was associated with later poor mental well‐being, and (2) poor mental well‐being at baseline was associated with later high weight status.

### 2.6 Quality Assessment

A quality assessment was conducted using the well‐known standardized critical appraisal instrument from the Joanna Briggs Institute regarding cohort studies [31]. The quality assessment was not used as a tool for further excluding of studies but rather as part of the analysis. Of the 11 items, four items were specified to fit the purpose of the current review. First of all, Items 1 and 2 (regarding differences in the two groups) were only relevant for case–control studies and therefore not included. Regarding Items 3 and 7 (‘Were exposure/outcomes measured in a valid and reliable way?’), the assessment was ‘yes’ if mental well‐being was assessed using validated tools, either self‐reported or proxy (parent or teacher). Weight status was assessed “yes” if objectively measured by trained staff and further handled with age‐ and gender‐specific standards: Center for Disease and Control and Prevention (CDC), World Health Organization (WHO), or International Obesity Task Force (IOTF). Weight status was assessed “no” if based on self‐reported data. Item 6 (“Were the groups/participants free of the outcome at the start of the study?”) was additionally assessed as “yes” if the outcome (weight status or mental well‐being) was measured at baseline and adjusted for. For Item 8 (“Was the follow‐up time reported and sufficient to be long enough for outcomes to occur?”), a follow‐up period of less than a year and longer than 10 years was assessed to be inappropriate.

The quality assessment was performed independently by two reviewers I.S.H and S. S, C.L.W. or I.R.H. Any disagreements between the reviewers were resolved through discussion between I.S.H and I.R.H, and if necessary, a third reviewer (C.L.W. or S.S.) was involved in the final agreements.

### 2.7 Protocol and Registration

This systematic review was registered at PROSPERO (no. 487603) on December 12, 2023.

### 2.8 Ethical Considerations

By Danish law, ethical authority approval for noninvasive medical research is not required.

## 3 Results

The literature search yielded 4158 potentially relevant studies. After removing duplicates, 2702 unique studies were screened. A total of 110 studies were identified as eligible; of these, 77 studies were cross‐sectional in design and were therefore excluded (see overview of the cross‐sectional studies in Table S1), while 33 studies with a longitudinal design were included in the present systematic review (Figure 1).

**FIGURE 1:**
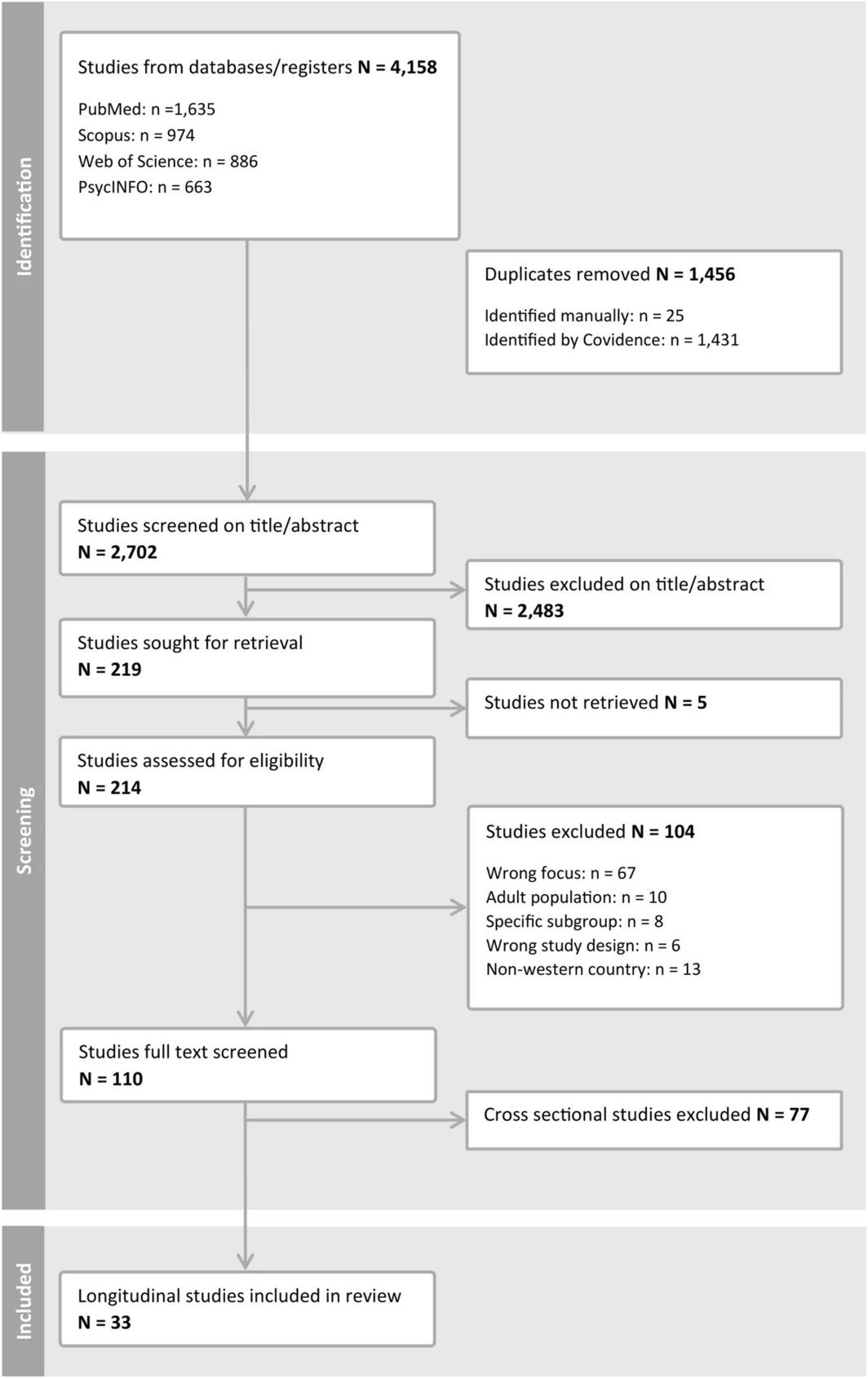
Flowchart of literature Legend for Figure 1: The original literature search was conducted on November 23, 2023. An updated search (rerun) was performed on January 10, 2025.

Of the 33 included studies, 18 investigated the association between weight status at baseline and mental well‐being at follow‐up (presented in Table 2), seven studies investigated the association of mental well‐being at baseline and weight status at follow‐up (presented in Table 3), and eight studies investigated the bidirectional association between weight status and mental well‐being (presented in both Tables 2 and 3). The included studies were published between 1994 and 2022. Most of the studies were based on data from the United States (*n* = 12) and Australia (*n* = 9) (Tables 2 and 3). Other studies were from the United Kingdom (*n* = 2), Spain (*n* = 2), Belgium (*n* = 1), Ireland (*n* = 2), the Netherlands (*n* = 2), New Zealand (*n* = 1), Norway (*n* = 1), and Switzerland (*n* = 1). Most of the studies included both boys and girls, whereas two included only girls [35, 39]. Most studies included school‐aged children at baseline, whereas fewer included children of preschool age, and no studies focused on children of late teen age. One study had no information on the specific age of the participants [41].

**TABLE 2:** Characteristics and results from included longitudinal studies examining associations between weight status and later mental well‐being.

Author, year (citation)	Title	Study population	Follow‐up period	Year of baseline data collection	Study location	Measurement of weight status	Measurement of well‐being	Adjustments	Results	Summary conclusion No., expected or unexpected association
**Self‐esteem**										
Rumpel and Harris 1994 [32]	The influence of weight on adolescent self‐esteem	*N* = 936, 48.1% girls Age: 9–11 years	6 years	1969–74 (year of birth 1960–1963)	The United States	Measured BMI. Reference: National sample. Continuous variable	Self‐reported self‐esteem: Adolescent self‐esteem and locus of control (eight items—unidentified tool)	Eating behavior, relative IQ, race, anxiety, sex, sociability, income, and school problems	No association between BMI and self‐esteem or BMI and locus of control	**No association**
Strauss 2000 [33]	Childhood obesity and self‐esteem	*N* = 1090, 51% girls Age: 9–10 years	4 years	1992 or 1994	Australia	Measured (~75%) and self‐reported (~25%) BMI. Reference: National Health and Nutrition Examination Survey. Categorized variable; (1) nonobesity < 95th percentile and (2) obesity = 95th percentile	Self‐reported self‐esteem: SPPC	Change in global self‐esteem. Stratified by sex and race	Obesity associated with decreased levels of self‐esteem across sex, race, and self‐reported/measured height and weight. Similarly, children with obesity had higher odds of low self‐esteem, compared to children without obesity	**Expected association**
Hesketh et al. 2004 [34]	Body mass index and parent‐reported self‐esteem in elementary school children: evidence for a causal relationship	*N* = 1157, 51% girls Age: 5–10 years	3–4 years	1997	Australia	Measured BMI. Reference percentile IOTH and *z*‐score CDC. Continuous and categorized variable: (1) nonoverweight, (2) overweight, and (3) obesity	Parent‐reported self‐esteem: Australia authorized adaption of the CHQ PF50 (six items regarding self‐esteem)	Baseline zBMI	Higher BMI *z*‐score associated with lower self‐esteem. Children with overweight/obesity had higher odds of low self‐esteem compared to children without overweight/obesity	**Expected association**
Biro et al. 2006 [35]	Self‐Esteem in Adolescent girls	*N* = 2379, 100% girls Age: 9–10 years	7–8 years	1994 (1998–2001 Phase 2)	The United States	Measured BMI. Reference: race‐ and age‐specific within the study population. Continuous variable. Categorized variable for interaction analyses; (1) “med BMI” = within one SD of the mean, (2) “low BMI” > one SD below the mean, and (3) “high BMI” > greater than one SD above the mean	Self‐reported self‐esteem: SPPC (first 4 years) and SPPA (from Year 5)	Age, race, and BMI, with parental education and clinical site as covariates, significant variables included BMI and interactions between age and race, race and BMI, and the triple interaction between age, race, and BMI	Higher BMI associated with lower self‐esteem. The association differs across age and race	**Expected association**
Wang et al. 2009 [36]	The influence of childhood obesity on the development of self‐esteem	*N* = 2879, 49.7% girls Age: 10–11 years	4 years	1994–1995	The United States	Parent‐reported BMI. Reference IOTF 2000. Categorized variable; (1) Normal weight, (2) overweight = 85th percentile, and (3) obesity = 95th percentile	Self‐reported self‐esteem: four items—unidentified tool	Sex, school performance, rural or urban residence, household income, parental education, physical activity, screen time, and baseline weight	Children with obesity had higher odds of low self‐esteem, compared to children normal weight. No difference in the odds of low self‐esteem between children with overweight and children with normal weight	**Expected association and no association**
Jeffery et al. 2014 [37]	Mood and its association with metabolic health in adolescents: a longitudinal study, EarlyBird 65	*N* = 208, 44.7% girls Age: 7 years	9 years	1999	Spain	Measured BMI. Reference UK 1990 BMI reference curve (SDS) Body fat measured with a DEXA‐scan. Continuous variables	Self‐reported mood: PANAS‐C	Sex, age at peak height velocity, physical activity, fat%, and metabolic risk *z*‐score	Increased body fat percentage from 7 to 16 years of age associated with lower mood. However, body fat percentage at age 7 was not associated with moos at age 16	**Expected association and no association**
Gibson et al. 2017 [38]	The psychosocial burden of childhood overweight and obesity: evidence for persisting difficulties in boys and girls	*N* = 212, 51% girls Age: 6–13 years	1–2 years	2004	The United States	Measured BMI. Reference CDC 2000. Continuous variable. Strata categories; (1) Healthy weight and (2) overweight/obesity = 85th/95th percentile	Self‐reported self‐esteem: SPPC and QoL: PedsQL	Age, maternal BMI, and family income. Stratified by sex and BMI category (overweight/obesity and healthy weigh)	Among children with overweight/obesity at baseline and across sex, BMI *z*‐scores associated with lower self‐esteem. No association between BMI *z*‐scores and self‐esteem among health weight children at baseline and across sex	**Expected association**
Kim and Fletcher 2021 [39]	The effects of relative body weight on socioemotional and schooling outcomes among female adolescents in the United States	*N* = 6748, 100% girls Age: 15.7 years (mean)	~1 year (Wave 2), 6 years (Wave 3), 13 years (Wave 4), and 22 years later (Wave 5)	1994–1995	The United States	Self‐reported BMI. Reference CDC 2016. Continuous and relative BMI in decile	Self‐reported self‐esteem: modified six items version of RSE	Race/ethnicity, age, standardized PVT score, firstborn, maternal education, family income, rural status, decile BMI, and school and grade fixed effects	Relative BMI associated with decreased self‐esteem	**Expected association**
Meland et al. 2021 [40]	How body concerns, body mass, self‐rated health, and self‐esteem are mutually impacted in early adolescence: a longitudinal cohort study	*N* = 1.225, 50% girls Age: 11–13 years	2 years	2011	Norway	Self‐reported weight and height. Continuous variable	Self‐reported self‐esteem: RSE	Age, family affluence (SES) and baseline self‐esteem. Stratified by sex	Higher BMI associated with lower self‐esteem among boys. No association between BMI and self‐esteem among girls	**Expected association and no association**
**Socioemotional skills**										
Datar and Sturm 2004 [41]	Childhood overweight and parent‐ and teacher‐reported behavior problems: Evidence from a prospective study of kindergartners	*N* = 9949, 42.9% girls Age: not available	2 years	1998–1999	The United States	Measured BMI. Reference CDC 2000. Categorized variable; (1) Not overweight and (2) Overweight = 95th percentile	Teacher‐ and parent‐reported Behavior problems: SRS	Race‐ethnicity, annual family income, whether the child belongs to a single‐parent household, maternal depression, parent–child interaction, and child's birth weight. Stratified by sex	No association between overweight and behavior problems across sex, race, and reporting (parent/teacher)	**No association**
Jansen et al. 2008 [28]	Feeling fat rather than being fat may be associated with psychological well‐being in young Dutch adolescents	*N* = 787, 49% girls Age: 9–10 years	3 years	2000–2001	The Netherlands	Measured (at baseline) and self‐reported (follow‐up): BMI. Reference IOTF 2000. Categorized variable: (1) Not overweight, (2) overweight BMI > 25, and (3) obesity BMI > 30	Self‐reported emotional symptoms: SDQ 5 item subscale	Sex, country of origin, and level of education	No association between BMI categories and emotional symptoms	**No association**
Griffiths et al. 2011 [42]	Is obesity associated with emotional and behavioral problems in children? Findings from the Millennium Cohort Study	*N* = 11,202, 49.9% girls Age: 3 years	2 years	2000–2002	The United Kingdom	Measured BMI. Reference IOTF 2000. Categorized variable: (1) Normal weight < 85th percentile, (2) overweight = 85th percentile, and (3) obesity = 95th percentile	Parent‐reported emotional and behavioral symptoms: SDQ	Child's ethnicity, maternal socioeconomic status, mother's highest academic qualification, maternal age at birth, household income, lone‐motherhood status, no. of children in the household, maternal treatment for depression or anxiety, maternal weight status, emotional/behavioral problems at age 3, and weight status at age 5. Stratified on sex	No association between BMI categories and any emotional or behavioral problems	**No association**
Jansen et al. 2013 [43]	Development of Mental Health Problems and Overweight Between Ages 4 and 11 Years: A Population‐Based Longitudinal Study of Australian Children	*N* = 3197, 49.3% girls Age: 4–5 years	6 years	2004	Australia	Measured BMI. Reference CDC, categorized by IOTF. Categorized variable: (1) normal weight (including underweight) and (2) overweight (including obesity)	Parent and teacher‐reported emotional and behavioral symptoms: SDQ	Sex, indigenous status, English spoken at home, SES based on parent‐reported education, occupational level, and family income categorized into quintiles, child age, and maternal stress	Repeated measures of overweight associated with higher levels of total difficulties, particularly peer problems and emotional symptoms. Similar findings between parent and teacher‐reported well‐being, though strongest for teacher reported. Compared to children with normal weight, children with overweight/obesity on average had higher total difficulty score, particularly for peer problems and emotional symptoms. Similarly, children with overweight/obesity had increased odds of having borderline/abnormal peer problems, compared to children with normal weight	**Expected association**
Michels et al. 2015 [44]	Cross‐Lagged Associations Between Children's Stress and Adiposity: The Children's Body Composition and Stress Study	*N* = 316, 50% girls Age: 5–10 years	1 and 2 years	2010–2012	Belgium	Measured BMI and WHtR, fat% (measured by BIA). Reference Cole et al. 1990. Continuous variable	Parent‐reported emotional and behavioral symptoms: SDQ	Sex, age, and parent education	Adiposity (zBMI, fat %, and WHtR) associated with higher stress, particularly negative emotions. External eating was a moderator of this association	**Expected association**
Pérez‐Bonaventura et al. 2015 [45]	The relationship between weight status and emotional and behavioral problems in Spanish preschool children	*N* = 611, 50,1% girls Age: 3 years	1 and 2 years	2009	The Netherlands	Measured BMI. Reference WHO 2006. Continuous and Categorized variable; (1) nonoverweight and (2) Overweight = 95th percentile	Parent‐reported emotional and behavioral symptoms: SDQ	SES and baseline SDQ	Higher zBMI was associated with higher mean scores in hyperactivity problems, peer relationships problems, and total difficulties at 1‐year follow‐up, but only associated with peer relationship problems at 2‐year follow‐up. Children with overweight had higher odds of hyperactivity compared to children with normal weight at 1‐year follow‐up, but not at year follow‐up	**Expected association**
Tillery et al. 2016 [46]	Relations between baseline and nonlinear longitudinal changes in children's body mass index and internalizing symptoms	*N* = 12,674, 49% girls Age: 5.6 years (mean)	8–9 years	1998–1999	The United States	No information on data collection: BMI. Reference CDC 2009 + 2012 (underweight). Categories; (1) underweight = < −2 SD, (2) normal weight = average weight, (3) overweight= > 1.5 SD, and (4) obesity = > +2 SD	Teacher‐reported internalizing problems: SRS	Stratified on sex and race	No association between zBMI and internalizing symptoms, or changes in zBMI and internalizing symptoms, across sex and race	**No association**
Slykerman et al. 2020 [47]	Physical Activity, Sleep, Body Mass Index, and Associated Risk of Behavioral and Emotional Problems in Childhood	*N* = 871, 50.1% girls Age: 7 years	4 years	2002–2004. Baseline for this study is at 7 years, year of birth is reported as 1995–1997	Spain	Measured BMI. Reference Cole 1990. Dichotomous variable. At baseline a BMI ≥ 17.92 was categories as overweight/obesity for boys and for girls a BMI ≥ 17.75 at follow‐up these cutoffs were ≥ 20.55 (boys) and ≥ 20.74 (girls)	Self‐ and parent‐reported emotional and behavioral symptoms: SDQ. Parent‐ and teacher‐reported CRS emotional and behavioral symptoms: CRS	Sex, birth weight, maternal smoking, occupational status, age mother left school, marital status at birth, average hours of sedentary activity at 11 years, and FSIQ at 11 years	No association between BMI categories and emotional or behavioral problems	**No association**
Zhou et al. 2022 [48]	Body mass index and internalizing symptoms from early childhood through early adolescence: Trend of co‐development and directionality	*N* = 17,552 children, 48.8% girls Age: 5.6 years (mean)	7 waves. 5 months from Wave 1 to 2, after that 1 year	2010–2011	The United States	Measured BMI. Reference: IOTF. Continuous variable	Teacher‐reported internalizing symptoms: SSRS	Sex, race, and household income, maternal and paternal, externalizing symptoms education, and occupational prestige scores (SES)	Higher BMI associated with higher internalizing scores	**Expected association**
**Health‐related quality of life (HRQoL)**										
Williams et al. 2011 [49]	Changes in body mass index and health‐related quality of life from childhood to adolescence	*N* = 960, 51.1% girls. Age: 8.4–13.8 years	5 years	2000	Australia	Measured BMI. Reference IOTF (percentile and CDC *z*‐scores). Continuous variable	Child‐ and parent‐reported HRQoL: PedsQL	Age, sex and quartile of SEIFA disadvantage index, and zBMI baseline	No association between zBMI and HRQoL (PedsQL)	**No association**
Gopinath et al. 2013 [50]	Adiposity adversely influences quality of life among adolescents	*N* = 1025, 43.3% girls Age: 12.7 years (mean)	5 years	2004–2005	Ireland	Measured BMI, Waist (cm), body fat% (leg‐leg bioimpedance analysis by Body Composition Analyzer). Reference IOTF 2000. Categories; (1) Not overweight, (2) Overweight = 85th percentile, and (3) obesity = 95th percentile	Self‐reported* HRQoL: PedsQL (no info about reporting)	Age, sex, and ethnicity	Children with persistent obesity, but not children with persistent overweight, on average had a lower physical summery score, compared to children without overweight/obesity. No association between BMI categories and psychosocial scores of PedsQL	**Expected association and no association**
Jansen et al. 2013 [51]	Bidirectional associations between overweight and health‐related quality of life from 4–11 years: Longitudinal Study of Australian Children	*N* = 3939, 48.7% girls. Age: 4–5 years	6 years	2004	Australia	Measured BMI. Reference CDC, categorized by IOTF. Categories: (1) normal weight (including underweight) and (2) overweight (including obesity)	Parent/caregiver‐reported HRQoL: PedsQL	Sex, age, indigenous status, English‐speaking background, and family socioeconomic position	Children with overweight/obesity on average had a lower PedsQL score compared to children without overweight. Dose–response relationship with increasing number of observations with overweight/obesity associated with lower PedsQL score	**Expected association**
Wynne et la. 2014 [52]	The relationship between body mass index and health‐related quality of life in urban disadvantaged children	*N* = 255, 50% girls Age: 7–12 years	1 year	Not available	New Zealand	Measured BMI. Reference WHO 2007. Categories (1) underweight, (2) normal weight, (3) overweight = +1 SD or 25 kg/m2, and (4) Obesity = +2 SD or 30 kg/m2. Underweight and normal weight were pooled in the analysis	Self‐reported HRQoL: Kidscreen‐27	Adjustments not specified. Tested for effect modification with sex, age, depressive symptoms, perceived social support, and family structure	Higher zBMI associated with lower HRQoL. Children with obesity on average had lower HRQoL compared to children with normal/underweight. The association was modified by age, where the association was strongest at the oldest measured age (Age 12). No difference in HRQoL score between children with overweight and children with normal/underweight	**Expected association**
Ortiz‐Pinto et al. 2020 [53]	Parental perception of child health status and quality of life associated with overweight and obesity in early childhood	*N* = 1869, 49,8% girls Age: 4 years	2 years	2012	Australia	Measured BMI. Reference and cutoff points from the WHO 2006: ≥ 1 SD = overweight/obesity and waist circumference (> 90th percentile for “Abdominal obesity” as by Fernández et al. 2004 + International Diabetes Federation guidelines)	Parent‐reported HRQoL: Kidscreen‐10	Sex, age, mother's educational level, family purchasing power, weekly hours of screen time and videogames, weekly hours of physical activity, breastfeeding, and parent‐perceived health status at age 4 years	Weight status change from overweight/obesity to normal weight was associated with higher HRQoL. No association between persistent overweight/obesity and HRQoL	**Expected association and no association**
Basterfield et al. 2022 [54]	Changes in children's physical fitness, BMI and health‐related quality of life after the first 2020 COVID‐19 lockdown in England: A longitudinal study	*N* = 178, 46.6% girls. Age: 8–10 years	1 year	2019	The United Kingdom	Measured BMI. Reference Cole et al. 1995 *z*‐scores. Continuous *z*‐score variable. Categories (1) underweight ≤ 2nd centile, (2) healthy weight > 2nd < 85th centile, (3) overweight ≥85th < 95th centile, (4) obesity ≥ 95th < 99.6th centile, and (5) severe obesity≥ 99.6th centile	Self‐reported HRQoL: Kidscreen‐27	Age, sex, and baseline BMI *z*‐score	Increase in zBMI associated with physical well‐being, but not psychological well‐being	**Expected association and no association**
**Multiple well‐being constructs**										
Wake et al. 2010 [55]	Comorbidities of overweight/obesity experienced in adolescence: Longitudinal study	*N* = 923, 49.6% girls Age: 5.0–10.7 years	3 and 8 years	1997	Australia	Measured BMI. Reference IOTF. Categories; (1) nonoverweight < 25 kg/m2, (2) overweight > 25 kg/m2 and < 30 kg/m2, and (3) obesity > 30 kg/m2. Further categorized according to timing and persistence of overweight/obesity; (1) Never = nonoverweight at all three waves, (2) childhood only = overweight/obesity on 1997 and/or 2000, but not 2005, (3) adolescence only = only overweight/obesity in 2005, and (4) persistent = overweight/obesity in one or both of the 1997 or 2000 waves, as well as in 2005	Self‐ and parent‐reported emotional and behavioral symptoms: SDQ and HRQoL: PedsQL	Sex, age in months, and SEIFA disadvantage index	Overweight/obesity throughout childhood and adolescence associated with lower physical summery score of PedsQL (No association with psychological summery)	**Expected association**
Sawyer et al. 2011 [56]	Four‐Year Prospective Study of BMI and Mental Health Problems in Young Children	*N* = 3363, 49% girls Age: 4–5 years	4 years	2004	Australia	Measured BMI. Reference: CDC 2000. Continuous variable	Teacher‐ and parent‐reported emotional and behavioral symptoms: SDQ and HRQoL: PedsQL	Maternal educational level, Mothers native language (other than English), mothers Kessler K6 score, child indigenous status, mother occupation, neighborhood disadvantage, and baseline SDQ or PedsQL	Higher BMI associated with higher scores of SDQ total difficulties, peer problems and emotional symptoms (both parent‐ and teacher‐report), as well as PedsQL total, physical functioning, and social scales. Similarly, larger increases in BMI were associated with increased a score above the “at risk” cutoff on the PedsQL total, physical functioning, and social scales	**Expected association**

Abbreviations: BMI = body mass index; CDC = Centers for Disease Control and Prevention; CHQ‐PF50 = Child Health Questionnaire—Parent Form 50; CRS = The Conner's Rating Scale; DEXA‐scan = dual‐Energy X‐ray absorptiometry scan; HRQoL = health‐related quality of life; IOTF = International Obesity Task Force; KIDSCREEN = 10/27 items health‐related quality of life questionnaire; PANAS‐C = The Positive Affect and Negative Affect Schedule—Child Form; PedsQL = pediatric quality of live inventory; QoL = quality of life; RSE = Rosenberg Self‐Esteem; SDQ = Strengths and Difficulties Questionnaire; SPPA/SPPC = The Harter Self‐Perception Profile for Adolescents/Children; SRS = Social Rating Scale—adapted from the Social Skill Rating System; PSS = Perceived Stress Scale; SSRS = Social Skills Rating System; WHO = World Health Organization; WHtR = Waist‐to‐height ratio.

**TABLE 3:** Characteristics and results from included longitudinal studies examining associations between mental well‐being and later weight status.

Author, year (citation)	Title	Study population	Follow‐up period	Year of baseline data collection	Study location	Measurement of weight status	Measurement of well‐being	Adjustments	Results	Summary conclusion No., expected or unexpected association
**Self‐esteem**										
French et al. 1996 [57]	Self‐esteem and change in body mass index over 3 years in a cohort of adolescents	*N* = 1278, 51.3% girls Age 12–15 years	3 years	*Not available*	The United States	Measured BMI. Reference: National norms for age and sex—WHO Guidelines (Himes & Dietz). Continuous and categorized variable; (1) nonoverweight and (2) overweight/obesity = 85th percentile	Self‐reported self‐esteem: SPPA	Baseline self‐esteem, baseline BMI, father's occupation, grade in school and pubertal status. Stratified by sex	No association between self‐esteem and BMI or BMI categories	**No association**
Hesketh et al. 2004 [34]	Body mass index and parent‐reported self‐esteem in elementary school children: evidence for a causal relationship	*N* = 1157, 51% girls Age: 5–10 years	3–4 years	1997	Australia	Measured: BMI. Reference percentile IOTH and *z*‐score CDC. Continuous and categorized variable: (1) nonoverweight, (2) overweight, and (3) obesity	Parent‐reported self‐esteem: Australian authorized adaption of the CHQ PF50 (six items regarding self‐esteem)	Baseline self‐esteem	No association between self‐esteem and zBMI. Children with low self‐esteem had higher odds for overweight/obesity	**No association and expected association**
Meland et al. 2021 [40]	How body concerns, body mass, self‐rated health and self‐esteem are mutually impacted in early adolescence: a longitudinal cohort study	*N* = 1225, 50% girls Age: 11–13 years	2 years	2011	Norway	Self‐reported weight and height. Continuous BMI‐variable	Self‐reported self‐esteem: RSE	Age, family affluence (SES), baseline BMI, and sex	Higher self‐esteem associated with lower BMI	**Expected association**
**Socioemotional skills**										
Mamun et al. 2005 [58]	Family and early life factors associated with changes in overweight status between ages 5 and 14 years: Findings from the Mater University Study of Pregnancy and its outcomes	*N* = 2188, ~47.7% girls. Aged 5 years	9 years	1986–1989	Australia	Measured BMI. Reference IOTF. 5‐year‐old child was defined as overweight/obesity if a child's BMI was greater than 17.42 kg/m2 for a boy and 17.15 kg/m2 for a girl. Overweight/obesity was defined at age 14 years if BMI exceeded 22.62 kg/m2 for a boy and 23.34 kg/m2 for a girl. Categorized variable for weight change; (1) normal BMI at ages 5 and 14 years, (2) normal BMI at age 5 years but overweight or obesity at 14 years, (3) overweight or obesity at ages 5 years but normal at 14 years, and (4) overweight or obesity at ages 5 and 14 years.	Parent‐reported behavioral problems: CBCL	Parental BMI, birth weight, gestational age, rate of weight gain during the first 6 months after birth, duration of breastfeeding, maternal depression, parental education, and family income	No association between behavioral problems and change in weight status	**No association**
Chang **and** Gable 2013 [59]	Predicting weight status stability and change from fifth grade to eighth grade: The significant role of adolescents' social–emotional well‐being	*N* = 6220, 51% girls Age: 10.25–12.77 years, 11.23 years (mean)	3 years	2004–2005	The United States	Measured BMI. Reference CDC 2000. Categories; (1) healthy weight (< 85th percentile), (2) overweight (85–95th percentile), and (3) obesity (> 95th percentile)	Teacher‐reported interpersonal skills: SRS modified version. Child‐reported self‐concept: The self‐description questionnaire	Parental reported of one's own health and child health, sex, race, age in months at fifth grade, highest parent education level in the household, family structure, and household poverty level	Socioemotional functioning was associated with weight status change	**Expected associations**
Graziano et al. 2013 [60]	Predicting weight outcomes in preadolescence: The role of toddlers' self‐regulation skills and the temperament dimension of pleasure	*N* = 195, 58.5% girls Age: 2 years	8 years	Not available	The United States	Measured BMI. Reference CDC 2000. Continuous variable and change in BMI	Parent‐reported behavioral symptoms: TBAQ	When BMI as outcome: Racial status and self‐regulation. When weight change as outcome: Racial status and 4‐year BMI	Self‐regulation skills and temperament dimension associated with BMI and obesity and weight change from ages 4 to 10 years	**Expected association**
Jansen et al. 2013 [43]	Development of Mental Health Problems and Overweight Between Ages 4 and 11 Years: A Population‐ Based Longitudinal Study of Australian Children	*N* = 3197, 49.3% girls Age: 4–5 years	6 years	2004	Australia	Measured BMI. Reference CDC, categorized by IOTF. Categorized variable: (1) normal weight (including underweight), and (2) overweight (including obesity)	Parent and teacher‐reported emotional and behavioral symptoms: SDQ	Sex, indigenous status, English spoken at home, SES based on parent‐reported education, occupational level, and family income, was categorized into quintiles. Child age, and maternal stress	No association total difficulties (SDQ) and zBMI (neither parent‐ nor teacher‐reported or analyzed with continuous variables or categorical variables). Repeated episodes of peer problems (parent and teacher reported) associated with increase in zBMI	**No associations and expected**
Jernigan et al. 2015 [61]	Emotional Health Predicts Changes in Body Mass Index (BMI z) Among Black and Latino Youth	*N* = 767, 55% girls Age: 10.8 years (mean)	2 years	2009	The United States	Measured BMI. Reference CDC 2000. Continuous variable	Self‐reported emotional symptoms: a 10‐item questionnaire adapted from HBSC	School clustering, BMI *z*‐score at Wave 1, sex, age, race/ethnicity, eligibility for free or reduced price lunch, and change in height from Wave 1 to Wave 2	Greater negative emotional symptoms associated with increased zBMI	**Expected association**
Michels et al. 2015 [44]	Cross‐Lagged Associations Between Children's Stress and Adiposity: The Children's Body Composition and Stress Study	*N* = 316, 50% girls Age: 5–10 years	1 and 2 years	2010–2012	Belgium	Measured BMI and WHtR, fat% (measured by BIA). Reference Cole et al. 1990. Continuous variable	Parent‐reported emotional and behavioral symptoms: SDQ	Sex, age, and parent education	No association with overall stress and adiposity (BMI, fat %, and WHtR). Subdomain of negative emotions associated with increased risk of adiposity	**No associations and expected association**
Tillery et al. 2016 [46]	Relations between baseline and nonlinear longitudinal changes in children's body mass index and internalizing symptoms	*N* = 12,674, 49% girls Age: 5.6 years (mean)	8–9 years	1998–1999	The United States	No information on data collection: zBMI. Reference CDC 2009 + 2012 (underweight). Categories; (1) underweight = < −2 SD, (2) normal weight = average weight, (3) overweight= > 1.5 SD, and (4) obesity = > +2 SD	Teacher‐reported internalizing problems: SRS	Stratified on sex and race	No association between internalizing symptoms and obesity among Black boys and Black/White girls. Among White boys internalizing symptoms associated with obesity	**Expected and no associations**
Donnchadha et al. 2020 [62]	Rethinking the association between overweight/obesity and adhd in children: A longitudinal and psychosocial perspective	*N* = 5263, 51.4% girls Age: 9 years	4 years	2007–2009	Ireland	Measured BMI. Reference IOTF. Categorized variable: (1) underweight, (2) healthy weight, and (3) overweight/obesity	Parent/caregiver‐reported emotional and behavioral symptoms: SDQ—three subscales: (1) Emotional symptoms, (2) conduct problems, and (3) hyperactivity/inattention	Sex, birth weight, maternal prenatal alcohol use, and maternal prenatal smoking	No association between SDQ domains and BMI	**No association**
Zhou et al. 2022 [48]	Body mass index and internalizing symptoms from early childhood through early adolescence: Trend of co‐development and directionality	*N* = 17552 children, 48.8% girls Age: 5.6 years (mean)	7 waves. 5 months from Wave 1 to Wave 2, after that 1 year	2010–2011	The United States	Measured BMI. Reference: IOTF. Continuous variable	Teacher‐reported internalizing symptoms: SSRS	Sex, race, and household income, maternal and paternal, externalizing symptoms education, and occupational prestige scores (SES)	Higher internalizing scores associated with higher BMI	**Expected association**
**Health‐related quality of life (HRQoL)**										
Williams et al. 2011 [49]	Changes in body mass index and health‐related quality of life from childhood to adolescence	*N* = 960, 51.1% girls Age: 8.4–13.8 years	5 years	2000	Australia	Measured BMI. Reference IOTF (percentile and CDC *z*‐scores). Categories; (1) not overweight, (2) overweight, and (3) obesity	Child‐ and parent‐reported HRQoL: PedsQL	Age, sex and quartile of SEIFA disadvantage, and BMIz baseline index	Lower parent‐reported (but not self‐reported) Total PedsQL score associated with zBMI	**Expected association**
Jansen et al. 2013 [51]	Bidirectional associations between overweight and health‐related quality of life from 4–11 years: Longitudinal Study of Australian Children	*N* = 3939, 48.7% girls Age: 4–5 years	6 years	2004	Australia	Measured BMI. Reference CDC, categorized by IOTF. Continuous variable	Parent/caregiver‐reported HRQoL: PedsQL	Sex, age, indigenous status, English‐speaking background, and family socioeconomic position	Low physical health was associated with higher BMI. No association with overall psychosocial health and BMI, but low social functioning (subdomain) was associated with higher BMI	**Expected and no associations**
Michels et al. 2016 [63]	Psychosocial Quality of Life, Lifestyle and Adiposity: A Longitudinal Study in Preschoolers (Ballabeina Study)	*N* = 291, 53% girls Age: 3.9–6.3 years, 5 years (mean)	10 months	2008–2009	Switzerland	Measured BMI, Waist‐to‐height ratio, and body fat % measured by BIA. Reference Cole et al. 1990. Continuous variable	Parent‐reported HRQoL: PedsQL	Parental education and migration status (SES), age, sex, parental physical activity (self‐reported), and mean parental BMI (self‐reported)	No association between total QoL score and adiposity (BMI, WHrR, or body fat %). Association between QoL and adiposity appeared after moderation by lifestyle factors: low psychosocial QoL associated with increased adiposity in children with unhealthy diet intake and high sedentary time. Low psychosocial QoL associated with decreased adiposity in children with high fruit intake and children with high physical activity level	**Expected and no associations**

*Note:* Measure of how many standard deviations below or above the population mean a raw score is.

Abbreviations: BMI = body mass index; CBCL = Child Behavior Checklist; CDC = Centers for Disease Control and Prevention; CHQ‐PF50 = Child Health Questionnaire—Parent Form 50; HBSC = The Health Behavior in School‐aged Children Study; HRQoL = Health‐Related Quality of Life; IOTF = International Obesity Task Force; PedsQL = Pediatric Quality of Live Inventory; QoL = quality of life; RSE = Rosenberg Self‐Esteem; SDQ = Strengths and Difficulties Questionnaire; SPPA = The Harter Self‐Perception Profile for Adolescents; SRS = Social Rating Scale—adapted from the Social Skill Rating System; SSRS = Social Skills Rating System; TBAQ = Toddler Behavior Assessment Questionnaire; WHO = World Health Organization; WHtR = Waist‐to‐height ratio; *Z*‐score = Also called a standard score.

### 3.1 Associations Between Weight Status at Baseline and Mental Well‐Being at Follow‐Up (*n* = 26)

In total, 26 studies examined the association between weight status at baseline and mental well‐being at follow‐up (Table 2).

Nine studies applied tools to assess self‐esteem [32, 33, 34, 35, 36, 37, 38, 39, 40]. Of these, eight studies found an association between weight status and later self‐esteem, in the expected direction as defined in the methods section [33, 34, 35, 37, 38, 39]. An expected association is, for instance, that a higher BMI *z*‐score was associated with lower self‐esteem or obesity increased the risk of low self‐esteem. Among the eight studies, one study found an expected association for obesity, but not overweight [36]. Another study indicated that increased body fat percentage in the follow‐up period, but not body fat percentage at baseline, was associated with lower self‐esteem [37]. Furthermore, one study found an expected association between weight status and self‐esteem among boys, but not among girls [40]. One study found no association between weight status and later self‐esteem [32].

Eleven studies measured aspects of socioemotional skills [28, 41, 42, 43, 44, 45, 46, 47, 48, 55, 56], with six studies finding an association in the expected direction between weight status at baseline and socioemotional skills at follow‐up [43, 44, 45, 48, 55, 56]. For instance, higher BMI was associated with total difficulties, internalizing symptoms, and peer relationship problems. Five studies found no association between weight status and socioemotional skills [28, 41, 42, 46, 47].

Eight studies measured HRQoL [49, 50, 51, 52, 53, 54, 55, 56], of which seven found higher weight status being associated with lower HRQoL [50, 51, 52, 53, 54, 55, 56]. Among these, Gopinath et al. found that children with persistent obesity, but not persistent overweight, had lower physical HRQoL summary scores [50]. Additionally, this study did not find an association between BMI categories and psychosocial HRQoL summary scores. Similar findings were reported by Basterfield et al. [54]. Another study found that children who changed weight status from overweight/obesity to normal weight had higher HRQoL at follow‐up, but persistent overweight/obesity in the follow‐up was not associated with HRQoL [53]. One study did not find an association (Table 2) [49].

### 3.2 Associations Between Mental Well‐Being at Baseline and Weight Status at Follow‐Up (*n* = 15)

In total 15 studies examined the association between mental well‐being at baseline and weight status at follow‐up (Table 3).

Three studies applied tools to assess self‐esteem [34, 40, 57]. Two studies [34, 40] found an expected association between self‐esteem at baseline and weight status at follow‐up. An expected association is, for instance, that lower mental well‐being at baseline is associated with higher weight status at follow‐up. Hesketh et al. found that children with low self‐esteem had higher odds of overweight/obesity but did not find an association between self‐esteem and BMI z‐scores [34]. Additionally, Meland et al. found that higher self‐esteem was associated with lower BMI [40]. One study did not find an association between self‐esteem at baseline and weight status at follow‐up [57].

Nine studies measured aspects of socioemotional skills [43, 44, 46, 48, 58, 59, 60, 61, 62]. Of these, seven studies found low socioemotional skills associated with higher BMI [43, 44, 46, 48, 59, 60, 61]. Jansen et al. found that repeated measures of the subdomain with peer problems were associated with higher BMI *z*‐scores [43]. However, there was no association with total difficulties from Strength and Difficulties Questionnaire (SDQ) and BMI *z*‐scores. Also, one study only found an association among White boys but not among White girls or Black boys or girls [46]. Also, another study found that the subdomain of negative emotions was associated with an increased risk of adiposity (BMI, fat percentage, and waist‐to‐height ratio), but no association with overall stress [44]. Two studies found no association [58, 62].

Three studies measured HRQoL [49, 51, 63]. All three studies found an expected association between HRQoL at baseline and weight status at follow‐up. However, one study only found an association of a low physical summary score and a low score on the subdomain of social functioning, with higher BMI, but not with the psychosocial summary score [51]. In another study, only parent‐reported HRQoL, but not self‐reported HRQoL, was associated with higher BMI [49]. Also, one study did not find an association between HRQoL and weight status in main analyses; however, in further analyses, an association appeared after moderation by lifestyle factors [63].

### 3.3 Quality Assessment

The included studies varied in quality, with no study obtaining maximum rating using the instrument from the Joanna Briggs Institute (Table 4).

**TABLE 4:** Quality assessment of included longitudinal studies.

Authur, year (citation)	3. Was the exposure measured in a valid and reliable way?	4. Were confounding factors identified?	5. Were strategies to deal with confounding factors stated?	6. Were the groups/participants free of the outcome at the start of the study (or at the moment of exposure)?	7. Were the outcomes measured in a valid and reliable way?	8. Was the follow‐up time reported and sufficient to be long enough for outcomes to occur?	9. Was follow‐up complete, and if not, were the reasons to loss to follow‐up described and explored?	10. Were strategies to address incomplete follow‐up utilized?	11. Was appropriate statistical analysis used?
**Weight status to mental well‐being**									
Rumpel and Harris 1994 [32]	Unclear	Yes	Yes	No	No	Yes	No	No	Yes
Strauss et al. 2000 [33]	No	No	No	Yes	Yes	Yes	Yes	No	Yes
Datar and Sturm et al. 2004 [41]	Yes	Yes	Yes	Yes	No	Yes	Yes	No	Yes
Biro et al. 2006 [35]	Unclear	Yes	Yes	Yes	Yes	Yes	Yes	No	Yes
Jansen et al. 2008 [28]	Yes	Yes	Yes	Yes	Yes	Yes	Yes	No	Yes
Wang et al. 2009 [36]	No	Yes	Yes	Yes	No	Yes	Yes	Yes	Yes
Wake et al. 2010 [55]	Yes	Yes	Yes	Unclear	Yes	Yes	No	No	Yes
Sawyer et al. 2011 [56]	Yes	Yes	Yes	Yes	Yes	Yes	No	No	Yes
Griffiths et al. 2011 [42]	Yes	Yes	Yes	Yes	Yes	Yes	No	Yes	Yes
Gopinath et al. 2013 [50]	Yes	Yes	Yes	No	Yes	Yes	No	No	No
Wynne et la. 2014 [52]	Yes	Unclear	No	Unclear	Yes	Yes	Unclear	No	No
Jeffery et al. 2014 [37]	Yes	Yes	Yes	No	Yes	Yes	No	No	No
Pérez‐Bonaventura et al. 2015 [45]	Yes	Yes	Yes	Yes	Yes	Yes	Yes	No	Yes
Gibson et al. 2017 [38]	Yes	Yes	Yes	Yes	Yes	Yes	Unclear	Unclear	Yes
Slykerman et al. 2020 [47]	Yes	Yes	Yes	No	Yes	Yes	Yes	No	Yes
Ortiz‐Pinto et al. 2020 [53]	Yes	Yes	Yes	No	Yes	Yes	No	No	Yes
Kim and Fletcher 2021 [39]	No	Yes	Yes	Yes	Yes	Yes	Yes	Yes	Yes
Basterfield et al. 2022 [54]	Yes	Yes	Yes	Yes	Yes	Yes	Yes	No	Yes
**Mental well‐being to weight Status**									
French et al. 1996 [57]	Yes	Yes	Yes	Yes	No	Yes	No	No	Yes
Mamun et al. 2005 [58]	Yes	Yes	Yes	Yes	Yes	Yes	Yes	No	Yes
Williams et al. 2005 [49]	Yes	Yes	Yes	Yes	Yes	Yes	Yes	No	Yes
Chang and Gable 2013 [59]	Yes	Yes	Yes	Yes	Yes	Yes	Unclear	Yes	Yes
Graziano et al. 2013 [60]	Yes	Yes	No	Yes	Yes	Yes	No	No	Unclear
Jernigan et al. 2015 [61]	Yes	Yes	Yes	Yes	Yes	Yes	Unclear	No	Yes
Michels et al. 2016 [63]	Yes	Yes	Yes	Yes	Yes	No	Unclear	Not applicable	Yes
Donnchadha et al. 2020 [62]	Yes	Yes	Yes	Yes	Yes	Yes	Yes	Yes	Yes
**Bidirectional**									
Williams et al. 2011 [49]	Yes	Yes	Yes	Yes	Yes	Yes	Unclear	No	Yes
Jansen et al. 2013 [51]	Yes	Yes	Yes	Yes	Yes	Yes	Yes	Yes	Yes
Jansen et al. 2013 [43]	Yes	Yes	Yes	Yes	Yes	Yes	No	Yes	Yes
Michels et al. 2015 [44]	Yes	Yes	Yes	Yes	Yes	Yes	Yes	Unclear	Yes
Tillery et al. 2016 [46]	Unclear	Yes	Yes	Yes	Unclear	Yes	Yes	Yes	Yes
Hesketh et al. 2004 [34]	Yes	Yes	No	Yes	Yes	Yes	Yes	No	Yes
Meland et al. 2021 [40]	No	Yes	Yes	Yes	No	Yes	Yes	Yes	Yes
Zhou et al. 2022 [48]	Yes	Yes	Yes	Yes	Yes	Yes	Yes	Yes	Yes

*Note:* Quality assessment was done using an adopted version of the checklist for cohort studies from the Joanna Briggs Institute.

The sample sizes varied from 178 to 17,552 participants, and seven studies had a sample size below 500 participants. Most of the studies were assessed to have measured exposure (*n* = 26/33) and outcome (*n* = 27/33) in a reliable way. Because this systematic review examines the bidirectional relationship between weight status and mental well‐being, both weight status and well‐being can be exposure and outcome. Weight status was objectively measured in about 80% of the included studies (*n* = 27) and self/parent‐reported in three studies. Additionally, one study used objectively measured weight and height at baseline and self‐reported at follow‐up, another study assessed weight status with both objective and self‐reported weight and height, and one study did not provide information on the procedure for weight status assessment. Furthermore, the classification of weight status varied greatly across the studies. Although weight status categories such as normal weight, overweight, and obesity were most commonly used, the reference growth charts and the cutoff points for applied categories differed. In some studies, overweight and obesity were combined in one category; in others, overweight and obesity were examined separately, and again in some, only obesity was examined. Moreover, the reference group in categorical analyses varied, containing either children classified as normal weight only, normal weight and underweight, or normal weight and overweight. Additionally, continuous measures such as BMI *z*‐score were commonly used. Few studies examined body fat by dual‐energy x‐ray absorptiometry (DEXA)‐scan or bioimpedance and other anthropometric measures such as waist circumference.

The majority of studies used validated and well‐known tools to assess mental well‐being, the most frequently being SDQ (*n* = 9) and Pediatric Quality of Life Inventory (PedsQL) (*n* = 7). Examples of other tools included the social rating scale (SRS), Rosenberg Self‐esteem Scale and KIDSCREEN.

All studies adjusted for some confounding factors; however, priori factors and reasons to include identified confounding factors were rarely stated. About two‐thirds of the studies included some adjustment for socioeconomic position such as maternal education or income. About 20% of the studies investigated sex differences in the examined associations, and no studies examined differences across socioeconomic positions.

Attrition was a consistent issue across studies, and only 10 studies utilized strategies for loss to follow‐up in the analyses. Table 4 shows the quality rating of each individual study.

## 4 Discussion

### 4.1 Overall Findings

This review summarizes existing evidence on the longitudinal and potentially bidirectional association between weight status and poor mental well‐being among children and adolescents.

The majority of the studies examining weight status at baseline and mental well‐being at follow‐up found significant associations and thus provide evidence that higher weight status is associated with later lower mental well‐being. The studies that did not find an association were primarily older—data collection conducted before 2004 [28, 32, 41, 47]. The most comprehensively identified study investigated prospective associations of weight status and mental well‐being among more than 17,500 boys and girls who were assessed seven times from kindergarten through fifth grade [48]. The study provided evidence of a reciprocal association between internalizing symptoms and BMI from 7 to 11 years of age, emphasizing the importance of interventions addressing both body weight and mental well‐being issues during middle childhood to mitigate their long‐term negative impacts. The majority of the studies using BMI categories examined overweight and obesity combined in one category, and only four studies differentiated between overweight and obesity. Wang et al. [36], Gopinath et al. [50], and Wynne et al. [52] found an association between obesity—but not overweight—at baseline and later poor mental well‐being. Future studies should investigate whether the association between weight status and mental well‐being is driven by obesity alone or if a dose–response relationship is present.

Fewer studies examined associations between mental well‐being at baseline and weight status at follow‐up. Yet, the current review shows some evidence for the importance of socioemotional skills at baseline and later weight status. For instance, Graziano et al. reported that regulation skills among 195 2‐year‐olds predicted both variation in BMI development and the development of childhood obesity 8 years later [60]. Only three studies examined the association between self‐esteem at baseline and later weight status, where two studies found that low self‐esteem was associated with a higher risk of developing obesity later on [34, 40]. One study of older publication date found no association between self‐esteem and later weight status. The studies on mental well‐being at baseline were primarily conducted in younger children, where worries and difficulties related to mental well‐being may not be as prevalent as later in adolescence. No studies were identified examining mental well‐being in late youth and later weight status. Thus, there is a need for further studies to examine the importance of low mental well‐being, especially in late teenage years, for later weight status.

Interpretation of these findings should also consider the measurement properties of the instruments used to assess mental well‐being in the included studies. The most frequently applied instruments were the SDQ and the PedsQL, both of which are widely used and supported by substantial psychometric evidence. Systematic reviews indicate that the SDQ demonstrates generally acceptable test–retest reliability, with higher stability observed for total difficulties scores compared with subscales, and some variation across respondent type, e.g., parent, teacher, self‐report [64, 65, 66]. The PedsQL likewise shows good overall reliability, including evidence of test–retest stability [67, 68], although this is less consistently reported than other reliability indices. Other instruments used less frequently have also demonstrated acceptable test–retest reliability in prior studies. For example, the Rosenberg Self‐Esteem Scale has shown good temporal stability across adolescent populations [69] and more recently tested among children [70], and lastly, KIDSCREEN instruments have demonstrated acceptable to good test–retest reliability across European samples [71]. However, estimates vary by age group, context, and follow‐up interval. Given the longitudinal design of the included studies, variability in measurement, particularly across subscales and respondent types, may introduce measurement error and attenuate associations between weight status and mental well‐being. At the same time, moderate test–retest reliability is not unexpected in measures of mental well‐being among children and adolescents, as these constructs are inherently dynamic and sensitive to developmental and contextual changes. Consequently, some degree of variability over time may reflect true change rather than measurement error. This may partly contribute to the heterogeneity observed across studies and should be considered when interpreting the magnitude and consistency of the reported findings.

### 4.2 Possible Mechanisms

Possible mechanisms underlying the found association of weight status on later mental well‐being include weight‐based stigmatization where social exclusion and discrimination are known to negatively influence the mental well‐being of children and adolescents [72, 73]. An increasing number of studies are investigating the importance of internalized weight stigma, referring to the process where public weight stigma is internalized resulting in self‐devaluation and the fear of enacted stigma resulting from one's identification with a certain stigmatized group [74]. Additionally, excess body fat has been shown to increase inflammatory markers [75] that again have been associated with poor mental health [75, 76]. Finally, physiological limitations related to body size—e.g., being unable to participate in sports—have been shown to negatively influence quality of life [77]. Conversely, regarding mechanisms driving the association between poor mental well‐being and later high weight status, research has mainly focused on an altered stress response. That is, poor mental health is correlated with higher levels of stress and cortisol secretion [78, 79], which in turn has been shown to activate storage of fat tissue [79] and increase appetite and emotional eating patterns related to weight gain [80]. One of the included studies in this review found cortisol to be a moderate effect modifier in the relationship between well‐being and later weight status [44]. Here, stress was positively associated with a high weight in children with high cortisol awakening patterns [44]. Thus, overall research has emphasized the role of biological, behavioral, and social factors for the bidirectional link between weight status and mental well‐being.

An additional important mechanism especially relevant for the relationship between weight status and mental well‐being concerns the psychological construct of body dissatisfaction. That is, a perceived discrepancy between the actual physical appearance of one's body and the desired or ideal appearance of the body [81]. Findings from several cross‐sectional and longitudinal studies suggest that body dissatisfaction plays an important mediating role in the association between weight status and later poor mental well‐being in both early and late adolescence [82, 83, 84]. In a longitudinal study based on data from the Netherlands, Jansen et al. concluded that feeling or perceiving the body as being overweight was more important for mental well‐being among adolescents than actually having overweight or obesity [28]. In fact, they did not find any significant direct association between actual weight status and later mental well‐being when body dissatisfaction was adjusted for [28]. Thus, one explanation for the sometimes inconsistent findings for the predictive role of obesity for later mental well‐being may be the mediating effect of body dissatisfaction. This also suggests that greater emphasis needs to be given to body perception concerns in the prevention of poor mental well‐being.

Finally, aspects of the family environment may independently affect children's BMI and their likelihood of developing both overweight and obesity, as well as poor mental well‐being, e.g., socioeconomic disadvantage and parental mental health (some also suggest parental/maternal weight status). Most of the included studies performed some level of adjustment for socioeconomic position. However, studies may still be confounded by socioeconomic factors or related factors such as adverse childhood experiences [85, 86]. Additionally, no studies investigated whether the association between weight status and mental well‐being differed between socioeconomic groups.

### 4.3 Public Health Prevention

The summarized evidence supports an integrated approach in which programs and interventions address both physical and psychosocial factors to promote children's health and well‐being, e.g., by incorporating mental well‐being, social relations, and supportive school or community environments into obesity prevention and treatment. However, the heterogeneity in methods used across studies may make it difficult to translate findings directly into prevention programs. For example, differences in BMI classifications across studies may make it difficult to translate directly into prevention programs. Some studies identify children with overweight and obesity as a vulnerable group, whereas others identify children with obesity alone, which has implications for screening practices and early identification. The same may apply for well‐being measures. Furthermore, well‐being is self‐reported, and since there has been increasing focus on mental health among children and youth in recent years, the association between weight status and mental well‐being may have changed over time. Lastly, the evidence in this review does not elucidate the mechanisms that explain the associations between weight status and mental well‐being, which would be helpful in designing targeted and effective public health programs and interventions.

### 4.4 Strengths and Limitations

A key strength of this review was the large number of studies included, which made the conclusions more nuanced. Additionally, the inclusion of longitudinal studies only allows comments on the causality between weight status and mental well‐being and vice versa. However, although we only included longitudinal studies, reverse causation in the identified associations cannot be ruled out as some studies did not exclude children/youth with either overweight/obesity or poor mental well‐being at baseline and simply adjusting for baseline status may not be sufficient.

The focus on the bidirectional association adds to the understanding of the complexities of the health issues and strengthens the relevance of the review to various research areas. Additionally, a key strength is the exclusive focus on mental well‐being rather than the somewhat broader term of mental health including clinical states, as done in previous reviews [10, 25]. We were mindful of the use of the SDQ in clinical screenings for ADHD and, as a result, paid close attention to ensuring that the studies focused on symptoms rather than formal diagnoses. Finally, the study selection, data extraction, and quality assessment of the current review were performed blinded and independently by at least two reviewers, decreasing the risk of bias.

The review has some limitations that need to be considered. First, some potentially eligible studies may have been missed. However, the broad search across four major databases (PubMed, PsycINFO, SCOPUS, and Web of Science) reduced the likelihood of overlooking relevant studies. Also, the search was rerun just before submission of the review. Second, there was substantial heterogeneity among the studies and their constructs, making a meta‐analysis impossible. Third, the inclusion criteria specified the inclusion of Western countries only, limiting the generalizability of our findings. Moreover, there was a notable predominance of studies from the United States and Australia, where high rates of childhood and adolescent overweight and obesity are observed [1]. Only one study was conducted in a northern European country [40]. Finally, no criteria for year of publication were defined. This was decided as no previous reviews, to our knowledge, have been conducted summarizing results on the longitudinal and bidirectional associations between weight status and mental well‐being in childhood and adolescence. Thus, the time period for the included studies (1994–2022) is wide, which may influence the results, given that the discourse and measurement of both weight status and mental well‐being have changed over time.

To summarize, overall findings suggested that higher weight status increased the risk of later poor mental well‐being, and there were indications of lower well‐being increasing the risk of higher BMI. However, fewer studies examined the direction from mental well‐being at baseline and later weight status. Furthermore, there was heterogeneity in designs and methods across included studies. Future research could focus on examining the importance of mental well‐being for later weight status, as well as underlying mechanisms including mediators and moderating factors.

## Conflicts of Interest

The Centre for Childhood Health was established by the Novo Nordisk Foundation and the Danish Ministry of the Interior and Health. The Novo Nordisk Foundation and the Danish Ministry of the Interior and Health have not been involved in the work with the current manuscript. Otherwise, the authors have no conflicts of interest to declare.

## Supporting information


**Table S1:** Characteristics and results from identified cross‐sectional studies examining associations between weight status and mental well‐being.

## Data Availability

Data sharing not applicable to this article as no datasets were generated or analysed during the current study.
