# Supplementary material for: Associations Between Weight Status and Mental Well‐Being in Childhood and Adolescence: A Systematic Review of Longitudinal Studies
Source: Obes Rev. 2026 Jun 9;27(12):e70169. doi: 10.1111/obr.70169 (PMC13749321; doi:10.1111/obr.70169)
Supplement: Supplementary file 1 [file OBR-27-e70169-s001.pdf]

Associations between weight status and mental well-being in childhood and adolescence: A systematic review of longitudinal studies

Stine Schramm[1], Iben Rask Heuck[1], Nina Rica Wium Geiker[1], Cathrine Lawaetz Wimmelmann[1]
[1]Centre for Childhood Health; Islands Brygge 41, 2300 Copenhagen S, Denmark
Corresponding author: Dr. Cathrine Lawaetz Wimmelmann, Centre for Childhood Health; Islands Brygge 41, 2300 Copenhagen S, Denmark, email: clw@cslt.dk

Supplementary table 1: Characteristics and results of identified cross-sectional studies examining associations between weight status and mental well-being

In total, 77 cross-sectional studies were identified (Table S1). Almost all studies used Body Mass Index (BMI) as a proxy for weight. The most frequently used measures for well-being were Health Related Quality of Life (HRQoL) (n=37), self-esteem (n=15), and socio-emotional factors (n=12). Other measures included self-efficacy (n=1) and life satisfaction (n=3). Ten studies included multiple constructs of well-being. Approximately two thirds of the cross-sectional studies found an expected association between weight and well-being; that is that a higher weight status was associated with poor well-being. Approximately 10 studies reported mixed results, while 12 found no association between weight and well-being.

Table S1: Characteristics and results from identified cross-sectional studies examining associations between weight status and mental well-being

Author, year (citation)	Title	Study population	Year data collection	Study location	Measurement weight	Measurement well-being	Results	Summary conclusion<br>No, expected or unexpected association
					**HRQoL**			
Wake et al., 2002 (1)	Parent-reported health status of overweight and obese Australian primary school children: a cross-sectional population survey	N = 2863, age 5-13 (M = 9.1), 49.5 % girls.	1997	Australia	BMI, measured: 1990 UK growth reference.	Health and well-being, parent reported: CHQ-PF.	Among boys, obesity was linked to poorer physical functioning, pain, mental health, self-esteem, general health, and parental impact (emotional and time), while overweight was linked to poorer role physical and mental health. Underweight was associated with more bodily pain. Among girls, obesity was linked to lower self-esteem and general health. Overweight was associated with poorer role emotional/behavioural health and self-esteem. Underweight was linked to poorer physical	Expected association

							functioning, role physical, general health, and emotional parental impact.	
Friedlander et al., 2003 (2)	Decreased Quality of Life Associated with Obesity in School-aged Children	N = 371, age 8-11 (M = 9.00), 49.9% girls.	Not available	United States	BMI, Measured: CDC. Independent.	HRQoL, parent reported: CHQ-PF50. Dependent.	Children with overweight vs. normal weight had lower score on the Psychosocial Health Summary, self-esteem, physical functioning, and parental emotional well-being. Children at risk for overweight vs. normal weight had lower physical functioning score. Children with underweight vs. Normal weight had lower self-esteem score.	Expected association
Pinhas-Hamiel et al., 2006 (3)	Health-related quality of life among children and adolescents: associations with obesity	N = 182 (Normal weight n= 94, obese n = 88), mean age 11.3-11.4 years. Approx. 55% girls.	Not available	Israel	BMI, measured: CDC. Independent.	HRQoL, child and parent reported: PedsQL. Dependent.	Higher BMI correlated with lower scores in physical, social, school, emotional and total HRQoL score.	Expected association
DeBeer et al., 2007 (4)	Health-related-quality-of-life in obese adolescents is decreased and inversely related to BMI	N = 93, aged 12-18 years.	2004 - 2005	Netherlands	BMI, case measured / control self-reported: Cole et al. (2000). Independent.	HRQoL, child reported: PedsQL & CHQ. Dependent.	PedsQL & BMI: Higher BMI correlated with lower scores in PedsQL total, physical health, psychosocial health scale scores, emotional functioning, and social functioning.<br>CHQ & BMI: Higher BMI correlated with lower scores in physical summary scale, global health, physical functioning, role/social limitations-behavioural, global behaviour, behaviour, mental health, self-esteem, general health perceptions, and family activities.	Expected association
Vissers et al., 2008 (5)	Overweight in Adolescents: Differences per Type of Education. Does One Size Fit All?	N = 994, age 16-18, 58.8% girls.	Not available	Belgium	BMI, measured: Cole et al. (2000).	HRQoL, child reported: SF-36 Health Survey.	Lower scores in HRQoL among females with obesity compared to normal and overweight. No significant differences were found in HRQL between obese, overweight, and normal weight boys.	Expected association & no association

Boyle et al., 2010 (6)	Physical activity, quality of life, weight status and diet in adolescents	N = 1,771, age 11-15 (M = 13.2), 48.3% girls.	2006 & 2007	England	BMI, self-reported: UK reference values.	HRQoL, child reported: PedsQL & EQ-5D.	Lower scores of QoL (PedsQL: physical functioning, emotional functioning, social functioning, psychosocial health summery score, total PedsQL & EQ-5D VAS) among children self-reporting BMI of overweight to obese compared to children reporting normal BMI status.	Expected association
Haraldstad et al., 2011 (7)	Predictors of health-related quality of life in a sample of children and adolescents: a school survey	N = 1,066, age 8–18, 54.03% girls.	Not available	Norway	BMI, measured: Cole et al. (2000). Independent.	HRQoL, child reported: KIDSCREEN-10. Dependent.	No association between BMI and HRQoL.	No association.
Keating et al., 2011 (8)	The health-related quality of life of overweight and obese adolescents – a study measuring body mass index and adolescent-reported perceptions	N = 2,890, age 11-18 (M = 14.6), 43.8% girls.	2005 - 2006	Australia	BMI, measured: IOTF.	HRQoL, self-reported: PedsQoL.	Higher weight status categories were associated with lower HRQoL scores. Normal weight vs. overweight: Total HRQoL, physical functioning and social functioning. Normal weight vs. obese: total HRQoL, physical functioning, social functioning and emotional functioning.	Expected association
van Grieken et al., 2012 (9)	Impaired parent-reported health-related quality of life of underweight and obese children at elementary school entry	N = 3,227, age 5-6 (M = 5.7), 50.7% girls.	2007 - 2010	Netherlands	BMI, measured: Cole et al. (2000 & 2007). Independent.	HRQoL, parent reported: CHQ-PF28. Dependent.	Higher weight status was associated with lower HRQoL scores. Compared to normal weight, severely obese individuals had poorer physical health, reduced physical functioning, and greater parental emotional and time impact. Obesity was linked to lower self-esteem and general health perception, while overweight was associated with reduced physical functioning. Underweight individuals reported poorer overall physical health, more bodily pain, and lower general health perception.	Expected association

Kesztyüs et al., 2013 (10)	Is central obesity associated with poorer health and health-related quality of life in primary school children? Cross-sectional results from the Baden-Wurttemberg Study	N = 1,888, age 5.4-9.8 (M = 7.1), 48.8% girls.	2010	Germany	BMI, measured: German reference. WHtR, measured: McCarthy and Ashwell.	HRQoL, parent reported: KINDLR & EQ5D-Y VAS.	Lower scores of HRQoL (EQ5D-Y VAS and KINDLR subscales: school and friends) among children with central obesity.	Expected association
Morales et al., 2013 (11)	Health-related quality of life, obesity, and fitness in schoolchildren: the Cuenca study	N = 1,158, age 8-11, 49.3% girls.	2010 - 2011	Spain	BMI, measured: Cole et al. (2000). Independent.	HRQoL, child reported: KIDSCREEN-52. Dependent.	Higher BMI were associated with lower scores of self-perception among females and with physical well-being, mood and emotions, autonomy and social support and peers when controlling for age. No association was found between weight categories and KIDSCREEN 10.	Expected association & no association
Petracci & Cavrini, 2013 (12)	The effect of weight status, lifestyle, and body image perception on health-related quality of life in children: a quantile approach	N = 4,338 (M = 13.8 years), 49.7% girls.	2007	Italy	BMI, measured: Cole et al. (2000). Independent.	HRQoL, child reported: EQ-5D-Y. Dependent.	Lower scores of HRQoL among children with overweight & obesity vs. normal weight.	Expected association
Chen et al., 2014 (13)	BMI, Health Behaviors, and Quality of Life in Children and Adolescents: A School-Based Study	N = 3,357. <u>Primary school</u>: M = 10.6. <u>High school</u>: M = 15.1. 51% girls.	2011 - 2012	Australia	BMI, measured: IOTF. Independent.	HRQoL, child reported: CHU9D. Dependent.	Lower HRQoL utility score among children with overweight & obesity vs. Normal weight in primary school.	Expected association
Cui et al., 2014 (14)	Health-related quality of life and body mass index among US adolescents	N = 7,031, age 12-17, 49% girls.	2001 - 2010	United States	BMI, measured: CDC. Independent.	HRQoL, child reported: CDC. Dependent.	Overweight and obesity was associated with lower odds of having excellent/very good health compared to normal weight. Also, obesity was associated with higher odds of fair/poor health that normal weight.	Expected association
Gandhi, 2015 (15)	Adolescent body weight and health-related quality of life rated by adolescents and parents: The issue of measurement bias Energy balance-related behaviors	N = 323, age 15-18 (M = 17.1), 51.7% girls.	2005	United states	BMI. Parent reported: CDC. Independent.	HRQoL, child and parent reported: PedsQL. Dependent.	There was no significant difference in HRQoL across weight categories.	No association

Helseth et al., 2015 (16)	A cross-sectional study of Health Related Quality of Life and body mass index in a Norwegian school sample (8–18 years): a comparison of child and parent perspectives	N = 1,066, age 8-18, 54.03% girls.	Not available	Norway	BMI, measured: IOTF. Independent.	HRQoL, parent and child reported: KIDSCREEN-52. Dependent.	Overweight was associated with lower physical well-being & self-perception.	Expected association
Parkinson et al., 2015 (17)	Influence of adiposity on health- related quality of life in the Gateshead Millennium Study cohort: Longitudinal study at 12 years.	N = 519, age 11-13 (M = 12), 51% girls.	1999 - 2000	England	FMI & waist circumference, measured. Independent.	HRQoL, child reported: KIDSCREEN-27. Weight-specific HRQoL, child reported: IWQOL-Kids. Dependent.	Higher FMI and waist circumference was associated with lower scores of physical well-being (KIDSREEN-27).<br>Higher FMI and waist circumference was associated with lower total score, body esteem, physical comfort, school life and family relations (IWQOL-Kids).	Expected association
Meyer et al., 2016 (18)	Health-related quality of life in children and adolescents: Current normative data, determinants and reliability on proxy-report	N = 530, age 9-18 (M = 13.2), 44.5% girls.	2012 - 2013	Germany	BMI	HRQoL, child and parent reported: KINDL-R.	No association between BMI and HRQoL.	No association
Mitchell & Steele, 2016 (19)	The Effect of Body Mass Index, Negative Affect, and Disordered Eating on Health-Related Quality of Life in Preadolescent Youth	N = 165, age 8-12 (M = 9.41), 50% girls.	Not available	United States	BMI, measured: CDC. Independent.	HRQoL, child reported: PedsQL. Dependent.	Higher BMI was associated with lower HRQoL.	Expected association
Andersen et al., 2017 (20)	Associations between health-related quality of life, cardiorespiratory fitness, muscle strength, physical activity and waist circumference in 10-year-old children: the ASK study	N = 1,129 children (M = 10.2), 52.08% girls.	Not available	Norway	Abdominal adiposity, measured: Brannsether et al. Independent.	HRQoL, child reported: KIDSCREEN-27. Dependent.	No association between abdominal adiposity and HRQoL.	No association

Muros et al., 2017 (21)	The association between healthy lifestyle behaviors and health-related quality of life among adolescents	N = 456, age 11-14 (M = 12.57), 51.5% girls.	2014	Spain	BMI, measured: Cole et al. (2000). Independent.	HRQoL, child reported: KIDSCREEN-27. Dependent.	Higher BMI was associated with lower HRQoL total score.	Expected association
Tsiros et al., 2017 (22)	Use-of-time and health-related quality of life in 10- to 13-year-old children: not all screen time or physical activity minutes are the same	N = 239, age 10-13. Boys: M = 12. Girls: M = 11.8. 44.8% girls.	2008 - 2009	Australia	Body fat % & BMI, measured: IOTF. Independent.	HRQoL, child reported: PedQL. Dependent.	Higher Body fat % was correlated with lower scores of physical, psychosocial, and total HRQoL.	Expected association
Halasi et al., 2018 (23)	Relationship between obesity and health-related quality of life in children aged 78 years	N = 182, age 7-8 (M = 7.71), 48.91% girls.	Not available	Serbia	BMI, measured: Cole et al. (2000). Body fat%, measured: Lobman et al.	HRQoL, parent reported: KIDSCREEN-27.	Among boys there was a significant difference in Social Support and Peers Domains with body fat % categorized as obesity vs. normal weight. Among girls there was no significant differences between any HRQoL domains and body fat %.	Expected association & no association.
Perez-Sousa et al., 2018 (24)	Fitness as mediator between weight status and dimensions of health-related quality of life	<u>Overweight/obesity:</u> N = 233, age 6-14 (M = 9.2), 46.4% girls. <u>Normal weight:</u> N = 105, age 7-14 (M = 10.8), 53.3% girls.	Not available	Spain	BMI, measured: Cole et al. (2000). Independent.	HRQoL, child reported: PedsQL & EQ-5D-Y VAS. Dependent.	Higher BMI is associated with lower score of HRQoL, especially in physical and psychosocial domains.<br>Among boys, significant differences were found in nearly all HRQoL domains, especially in physical and psychosocial functioning.<br>Among girls, similar patterns were observed, though differences in social functioning and VAS scores were not statistically significant.	Expected association
Hoare et al., 2019 (25)	Associations between combined overweight and obesity, lifestyle behavioural risk and quality of life among Australian regional school children: baseline findings of the Goulburn Valley health behaviours monitoring study	N = 809, age 9-12, 52.5% girls.	2016	Australia	BMI, measured: IOTF. Independent.	HRQoL, child reported: PedsQoL. Dependent.	Overweight/obesity was associated with lower levels of physical and global functioning compared to normal weight among boys. No association between BMI and HRQoL among girls.	Expected association & no association.

Bottolfs et al., 2020 (26)	Resilience and lifestyle-related factors as predictors for health-related quality of life among early adolescents: a cross-sectional study	N = 611 (M = 13.2), 49.1% girls.	2017	Norway	BMI, measured: IOTF. Independent.	HRQoL, child reported: KIDSCREEN-27. Dependent.	Higher BMI was associated with lower level of physical will-being.	Expected association
Killedar et al., 2020 (27)	Weight status and health-related quality of life during childhood and adolescence: effects of age and socioeconomic position	N = 4,083, age 4-17 years.	2004	Australia	BMI, measured: WHO. Independent.	HRQoL, parent reported: PedsQL. Dependent.	Overweight and obesity was associated with lower scores of HRQoL. Results also show that the association got stronger among older children.	Expected association
Cam & Top, 2020 (28)	Overweight, Obesity, and Health-Related Quality of Life Among high-school students in Turkey.	N = 1,216, age 14–18 (M = 15.58), 54.3% girls.	2018 - 2019	Turkey	BMI, measured: WHO.	HRQoL, child reported: SF-12.	Children with overweight reported significantly lower levels of physical and mental health compared to children with normal weight. Children with obesity reported significant lower levels physical health and mental health compared to both children with normal weight and overweight.	Expected association
Meixner et al., 2020 (29)	Health-related quality of life in children and adolescents with overweight and obesity: results from the German KIGGS survey	N = 1,771, age 11-17 (M = 13.85), 52.9% girls.	2014 - 2017	Germany	BMI, measured: German national reference. Independent.	HRQoL, child reported: KIDSCREEN-27. Dependent.	Overweight and obesity was associated with lower levels of physical well-being compared to normal weight among both girls and boys. Among boys obesity was further associated with lower levels of psychological well-being and parent-related well-being compared to normal weight.	Expected association
Thumann et al., 2020 (30)	Cross-sectional and longitudinal associations between psychosocial well-being and cardiometabolic markers in European children and adolescents	N = 6,519, age 6-15 (M =11.3), 50,5% girls.	2013 & 2014	Belgium, Cyprus, Estonia, Germany, Hungary, Italy, Spain and Sweden.	Waist circumference, measured. Dependent.	HRQoL, parent reported (<12 years): KINDLR. Independent.	Higher waist circumference was associated with lower scores of well-being.	Expected association

Mastorci et al., 2021 (31)	Relationship between Weight Status and Health-Related Quality of Life in a Sample of Early Adolescents from Central and Northern Italy: A Cross-Sectional Study of the AVATAR Project Participants	N = 1,707, age 10-14 (M = 12.5), 51% girls.	2017 - 2018	Italy	BMI, measured: IOTF.	HRQoL, child reported: KIDSCREEN-52.	Among Girls: Significant higher physical well-being, mood emotion, and self-concept in underweight vs. obesity. Significant higher autonomy in underweight vs. overweight. Significant higher self-perception in normal weight vs. Overweight. Among boys: Significant higher physical well-being and self-perception in Underweight vs. obesity. Significant higher physical well-being in normal weight vs. overweight and obesity. Further significant higher self-perception in normal weight vs. obesity.	Expected association
Hassan et al., 2021 (32)	Somatic complaints as a mediator in the association between body mass index and quality of life in children and adolescents	N = 733, age 2-18 (M = 8.2).	2010 - 2013	Netherlands	BMI, measured: WHO. Independent.	QoL, parent reported (age 2-9): PedsQL. Dependent.	Higher BMI was associated with lower QoL-score.	Expected association
Masini et al., 2021 (33)	The Determinants of Health-Related Quality of Life in a Sample of Primary School Children: A Cross-Sectional Analysis	N = 151, age 6-10 (M = 7.77), 45.8% girls.	2019	Italy	BMI, measured: Cole et al. (2007 & 2012). Independent.	HRQoL, child and parent reported: PedsQL. Dependent.	No association between BMI and HRQoL.	No association
Papadaki et al., 2022 (34)	Anthropometric, Lifestyle Characteristics, Adherence to the Mediterranean Diet, and COVID-19 Have a High Impact on the Greek Adolescents' Health-Related Quality of Life	N = 2,088, age 12-18 (M = 15.1), 50.7 % girls.	2021	Greece	BMI, self-reported: IOTF. Independent.	HRQoL, child reported: KIDSCREEN-27. Dependent.	Higher BMI was associated with lower HRQoL.	Expected association

Alanne et al., 2023 (35)	Elementary School-Aged Children's and Parents' Report of Health-Related Quality of Life and Relationships with Lifestyle Measures: A Cross-Sectional Study	N = 270, age 6-13 (M = 9.7), 47% girls.	2017 - 2018	Finland	BMI, measured: Finnish growth reference.	HRQoL, child and parent reported: PedsQL.	Significant higher scores in physical health, psychosocial health & total score in normal weight vs. Obesity. Significant higher scores in psychosocial health and total scores in normal weight vs. Overweight.	Expected association
Ródenas-Munar et al., 2023 (36)	Perceived Quality of Life Is Related to a Healthy Lifestyle and Related Outcomes in Spanish Children and Adolescents: The Physical Activity, Sedentarism, and Obesity in Spanish Study.	N= 3,534, age 8-16 years (mean age 13 (2.36) and 12.14 (2.28). 51.7% girls.	2019-2020	Spain	BMI and Waist-to-hight ratio. Ref. Fernandes et al. (2011). BMI: Categorized; 1) Under nutrition, 2) underweight, 3) Normal weight, 4) Overweight, and 5) Obesity. Waist-to-hight ratio: Dichotomized; abdominal obesity >= 0.5.	EQ-5D-5L (EuroQoL-5 Dimensions-5-Levels). Dichotomized; low HRQoL<=50 percentile, high HRQoL>50 percentile.	Significant lower odds for normal weight and higher odds for having overweight and abdominal obesity if low HRQoL compared to high HRQoL (adjusted).	Expected association
Rodrigues et al., 2023 (37)	Body size, form, composition, and a healthy lifestyle associates with health-related quality of life among Portuguese children	N = 1,333, mean age= 9.05. 51.2% girls.	2016 - 2017	Portugal	Waist and upper arm circumference, skinfold thickness, WHtR, Body fat% & BMI, measured: IOTF.	HRQoL, child reported: KIDSCREEN-27.	Children with higher BMI and body fat reported lower HRQoL, particularly in the physical well-being dimension of the KIDSCREEN-27 questionnaire. Among boys, BMI, waist circumference, waist-to-height ratio, and subscapular and suprailiac skinfolds showed the strongest associations with well-being. For girls, the strongest correlations were found with waist circumference and subscapular skinfold thickness.	Expected association
				**Self-esteem**				
Phillips & Hill, 1998 (38)	Fat, plain, but not friendless: self-esteem and peer acceptance of obese pre-adolescent girls	N = 313, age 9.1-10.8 (M = 9.10), 100% girls.	Not available	England	BMI, measured: Himes & Dietz.	Self-esteem, child reported: SPPC.	Significant lower scores in physical appearance and athletic competence in overweight & obese vs. normal weight.	Expected association

Pesa et al., 2000 (39)	Psychosocial Differences Associated with Body Weight Among Female Adolescents: The Importance of Body Image	N = 3204. Overweight / obesity: M = 16.3. Normal/underweight: M = 15.9. 100% girls.	1994 - 1995	United States	BMI, self-reported. Independent.	Psychosocial factors, child reported: Add Health Survey. Dependent.	Overweight/obesity was associated with lower self-esteem compared to normal weight.	Expected association
Gordon-Larsen, 2001 (40)	Obesity-Related Knowledge, Attitudes, and Behaviors in Obese and Non-obese Urban Philadelphia Female Adolescents	N = 64, age 11-15. Obesity: M = 13.56. Non-obesity: M = 13.59. 100% girls.	Not available	United States	BMI & triceps skinfold, Measured: NHANES I.	Self-concept / Self-esteem, child reported: PHCSCS.	No association between BMI and self-esteem.	No association
Mirza et al., 2005 (41)	Body dissatisfaction, self-esteem, and overweight among innercity Hispanic children and adolescents	N = 113 children, age 10-18.	2002	United States	BMI, measured: Barlow & Dietz. Independent.	Self-esteem, child reported: HSS. Dependent.	No association between BMI and self-esteem.	No association
Allen et al., 2006 (42)	Why do some overweight children experience psychological problems? The role of weight and shape concern	N = 207, age 7-13 (M = 10.1), 55.6% girls.	2004	Australia	BMI, measured: CDC. Independent.	Self-esteem, child reported: SPPC. Dependent.	Higher BMI was associated with lower scores of global & appearance self-esteem.	Expected association
Kristjánsson et al., 2010 (43)	Health Behavior and Academic Achievement Among Adolescents: The Relative Contribution of Dietary Habits, Physical Activity, Body Mass Index, and Self-Esteem	N = 5.810, age 14-15, 51.7% girls.	2000	Iceland	BMI, self-reported. Independent.	Self-esteem, child reported: RSES. Dependent.	Higher BMI was associated with lower self-esteem.	Expected association
McCullough et al., 2009 (44)	Self-perception in overweight and obese children: a cross-sectional study	N = 211, age 8-9, 46 % girls.	Not available	Ireland	BMI, measured: Cole et al. (2000). Independent.	Self-perception, child reported: SPPC. Dependent.	Higher BMI was associated with lower scores on social acceptance and physical appearance.	Expected association
Danielsen et al., 2012 (45)	Factors Associated with Low Self-Esteem in Children with Overweight	N = 4.167, age 11-13 (M = 11.8), 52% girls.	2006	Norway	BMI, self-reported: IOTF. Independent.	Self-esteem, child reported: SPPC. Dependent.	Higher BMI was associated with lower scores on physical appearance.	Expected association

Willows et al., 2013 (46)	High adiposity is associated cross-sectionally with low self-concept and body size dissatisfaction among indigenous Cree schoolchildren in Canada	N = 78 (M = 10.85), 64.10% girls.	2004	Canada	BMI, measured: WHO.	Self-concept, child reported: PHCSCS-2.	Higher BMI was associated with lower scores on global self-concept, intellectual and school status, and physical appearance and attributes.	Expected association
Wong et al., 2014 (47)	Body weight has no impact on self-esteem of minority children living in inner city, low-income neighbourhoods: a cross-sectional study	N = 910, age 9-12 (M = 10.3), 48.1% girls.	2009 - 2012	United States	BMI, measured: CDC.	Self-esteem, child reported: SPPC.	No association between BMI and self-esteem.	No association
Altıntaş et al., 2014 (48)	The role of physical activity, body mass index and maturity status in body-related perceptions and self-esteem of adolescents	N =1012, age 11-18 (M = 14.88), 48.02% girls.	Not available	Turkey	BMI, measured: Bioelectrical Impedance Analyser (TBF300, Tanita). Independent.	Self-esteem, child reported: RSES. Dependent.	Higher BMI was associated with lower self-esteem among girls. No association among boys.	Expected association and no association.
Bauer et al., 2017 (49)	Socioenvironmenta, Personal, and Behavioral Correlates of Severe Obesity among an Ethnically/Racially Diverse Sample of US Adolescents	N = 2706 (M = 14.3), 53.66% girls.	2009 - 2010	Minnesota	BMI, measured: CDC.	Self-esteem, child reported: EAT 2010 survey.	Significant lower scores in self-esteem among children with severe obesity compared to children with normal weight, overweight and class I obesity.	Expected association
Guillamón et al., 2019 (50)	Weight status, physical activity and self-concept in primary school children	N = 103, age 8-12 (M = 9.94), 61.2% girls.	2016 - 2017	Spain	BMI, Measured: Cole et al. (2000).	Self-concept, child reported: PHCSCS.	Significant lower scores in behavioural self-concept among children with overweight/obesity compared to normal weight.	Expected association
Vassiloudis & Costarelli, 2019 (51)	Excess body weight and abdominal obesity in relation to selected psychosocial characteristics in primary school children	N = 528, age 10-12, 51.5% girls.	2010	Greece	BMI, measured: IOTF. Waist circumferences, measured: NHANES.	Self-perception/self-esteem, child reported: SPPC.	Higher BMI & abdominal obesity was correlated with lower scores of scholastic competences, relations with peers, athletic competence, physical appearance, behavioural conduct, and global self-esteem.	Expected association

Blanco et al., 2020 (52)	Psychological well-being and weight-related teasing in childhood obesity: a case–control study	N = 100, age 8-12 (M = 10.14), 40% girls.	Not available	Spain	BMI, measured: Cole et al. (2000). Independent.	Self-esteem, child reported: LAWSEQ. Dependent.	Obesity was associated with lower levels of self-esteem compared to normal weight.	Expected association
				**Self-efficacy**				
Altan & Bektas, 2017 (53)	The effects of parental feeding styles, children's self-efficacy and social anxiety on adolescent obesity	N = 649 (M = 11.58), 55% girls.	2015	Turkey	BMI, measured: CDC (obesity only). Dependent.	Self-efficacy, child reported: The middle school self-efficacy scale. Independent.	Obesity was association with lower levels of interpersonal relations & preventive health care among boys, and preventive health care among girls.	Expected association
				**Socio-emotional skills**				
Sawyer et al., 2006 (54)	Is There a Relationship Between Overweight and Obesity and Mental Health Problems in 4- to 5-Year-Old Australian Children?	N = 4983, age 4-5 (M = 56.9 months), 49.1% girls.	2004	Australia	BMI, measured: IOTF.	Mental health problems, parent and teacher reported: SDQ.	Among boys there was significant differences between children with obesity, overweight, & normal weight in teacher-reported total difficulties and conduct problems. Among girls there was significant differences between children with obesity overweight, & normal weight in parent-reported peer problems.	Expected association
Pitrou et al., 2010 (55)	Child Overweight, associated psychopathology, and Social Functioning: A French School-based Survey in 6- to 11-year-old Children	N = 1030, age 6-11, 50.4 % girls.	2004 - 2005	France	BMI, self-reported: IOTF. Dependent.	Psychopathology and social functioning, child and parent reported: DI & SDQ. Independent.	Children with overweight correlated with significantly higher in parent-reported peer difficulties.	Expected association

Pastor & Reuben, 2011 (56)	Emotional/behavioral difficulties and adolescent obesity: effect of sex and Hispanic origin/race	N = 11.042, age 12-17, 48.4% girls.	2001, 2003 & 2004	United States	BMI, self-reported: CDC. Dependent.	Emotional/behavioural difficulties, parent reported: SDQ. Independent.	Among Hispanic (Spanish) girls' obesity was positively associated with emotional symptoms. In contrast, Hispanic girls (English) showed positive associations between obesity, and both conduct problems and peer problems. Among non-Hispanic white girls, obesity was linked to emotional symptoms, conduct problems, and peer problems. For Hispanic boys (English), a positive association was found between obesity and peer problems, while non-Hispanic white boys showed associations with emotional symptoms and conduct problems.	Expected association
Lu et al., 2012 (57)	Weight and Mental Health Status in Massachusetts, National Survey of Children's Health, 2007	N = 827, age 10-17, 49.3% female.	2007 - 2008	United States.	BMI, self-reported: CDC. Independent.	Mental health status, parent reported: NSCH survey. Dependent.	Overweight/obese was associated with higher levels of negative emotions compared to normal weight. No association among boys.	Expected and no association
Halfon et al., 2013 (58)	Associations Between Obesity and Comorbid Mental Health, Developmental, and Physical Health Conditions in a Nationally Representative Sample of US Children Aged 10 to 17	N = 43.297, age 10-17, 47.8% girls.	2007	United States	BMI, self-reported: CDC. Independent.	Psychosocial functioning, parent reported: NSCH survey. Dependent.	Obesity was associated with higher scores of internalizing problems, externalizing problems, grade repetition, school problems, and missed school days compared to normal weight. Overweight was associated to higher scores of externalizing problems compared to normal weight.	Expected association.
Koziel et al., 2017 (59)	Relationship between temperament and fatness in 11-year-old children and 17-year-old adolescents from Wrocław, Poland	Children: N = 122, age 11, 53.3% girls. Adolescents: N = 153, age 17, 58.2% girls.	2009	Poland	BMI & skin folds, measured: Lohman et al. Dependent.	Psychological/temperament factors, parent and child reported: EAS-C & EAS-D. Independent.	Among 11-year-old boys BMI was significant association with activity-factors, whereas sum of skinfolds was significant association with factors of activity, sociability, and shyness. Among 17-year-old boys there was only association between sum of skinfolds and activity-factors. Among 11-year-old girls BMI was	Expected and no association

							significant association with factors of emotionality. No associations were found among 17-year-old girls.	
Noonan & Fairclough, 2019 (60)	Cross-sectional associations between body mass index and social–emotional wellbeing among differentially active children	N = 6,011, age 7, 51,1% girls.	2007 - 2008	England	BMI, measured: Cole et al. (2000).	Social–emotional well-being difficulties, parent reported: SDQ.	Higher BMI was associated with higher scores of peer problems and total difficulties among girls. Higher BMI was associated with higher scores of peer problems, conduct problems, emotional problems, prosocial problems and total difficulty score.	Expected association.
Åvitsland et al., 2020 (61)	The association between physical fitness and mental health in Norwegian adolescents	N = 1,486, age 13-14 (M = 13.9), 50.6% girls.	2017	Norway	BMI, measured. Independent.	Psychological difficulties, child reported: SDQ. Dependent.	BMI: No association (total difficulties score).	No association
Bjertnaes et al., 2020 (62)	A Cross-Sectional Study of the Relationship Between Mental Health Problems and Overweight and Obesity in Adolescents	N = 3,196, age 15-16. 2002: M = 15.9, 49.3% girls. 2017: M = 15.8, 52.1% girls.	2002 & 2017	Norway	BMI, self-reported: Norwegian national reference. Dependent.	Mental health problems, child reported: SDQ. Independent.	Higher BMI was associated with higher scores of conduct problems among boys. Among girls the higher BMI was associated with higher scores of peer problems & emotional symptoms.	Expected association
Lundgren et al., 2021 (63)	Hyperactivity is associated with higher fat-free mass and physical activity in Swedish preschoolers: A cross-sectional study	N = 305 (M = 4.5), 46% girls.	2014 - 2015	Sweden	BMI, fat mass index & fat-free mass index, measured. Dependent.	Physiological strengths and difficulties, parent reported: SDQ. Independent.	Higher fat-free mass was associated with higher scores of hyperactivities. No association between fat mass and hyperactivity.	Unexpected and no association.
López-Gil et al., 2022 (64)	Role of Diet Quality in the Association Between Excess Weight and Psychosocial Problems in a Large Sample of Children in Spain	N = 3,772, age 4-14 (M = 9.5 years), 49.4% girls.	2017	Spain	BMI, self-reported: IOTF. Independent.	Psychosocial problems, parent reported: SDQ. Dependent.	Higher excess weight was associated with higher scores of psychosocial problems.	Expected association
Chen 2024 et al. (65)	Dose-Dependent Association Between Body Mass Index and Mental Health and Changes Over Time.	N = 1.036.869, age 11-15 (M = 13.55). 50.9 % girls.	2002-2018	Europe and North America	BMI z-score: WHO. Independent.	Psychosomatic concerns. Child reported: HBSC survey. Dependent.	Low body mass and overweight or obesity were associated with increased psychosomatic concerns compared to healthy weight. Underweight were associated with fewer psychosomatic concerns compared to healthy weight.	Expected association

				**Life satisfaction**				
Marques & De Matos, 2016 (66)	Trends in prevalence of weight and obesity: are Portuguese adolescents still increasing weight?	N = 8,610, age 11-17 (M = 13.8), 51.9% girls.	2002, 2006 & 2010	Portugal	BMI, self-reported: IOTF & WHO.	Life satisfaction, child reported: Cantril Self-Anchoring Striving Scale.	Higher BMI was associated with lower life satisfaction among Generation 1 (11–13 years in 2002). No association among Generation 2.	Expected association and no association.
Chai & Xue 2022 (67)	Weight, Weight Perceptions, and Health and Well-Being Among Canadian Adolescents: Evidence From the 2017-2018 Canadian Community Health Survey	N = 8.081, age 12-17. 49.4% girls.	2017-2018	Canada	Weight: WHO. Independent.	Mental health and life satisfaction. Child reported: CCHS survey. Dependent.	There was no association between weight categories and mental health or life satisfaction.	No association
Trombetta 2024 (68)	There is a correlation between nutritional status, Self-Rated Health and Life Satisfaction? Evidence from 2018 Health Behaviour in School-aged Children cross sectional study in a sample of Italian adolescents living in Tuscany Region.	N = 2760, age 11, 13 and 15. 98.9% girls.	2018	Italy	BMI z-score. Self-reported: WHO. Independent.	Life satisfaction. Child reported: HBSC survey. Dependent.	There is a statistically significant difference in all categories between boys and girls aged 13 and 15 years; in girls aged 11 and 13 years, the Life Satisfaction of the overweight group is statistically lower than that of normal and underweight groups.	Expected association
				**Multiple well-being constructs**				
Swallen et al., 2005 (69)	Overweight, Obesity, and Health-Related Quality of Life Among Adolescents: The National Longitudinal Study of Adolescent Health	N = 4743, age 12-20 (M = 16.03), 51,84% girls.	1996	United States	BMI, measured: CDC. Independent.	HRQoL, child reported: PedsQL. Self-esteem, child reported: RSES. Dependent.	Overweight and obesity was associated with poorer general health and more functional limitations compared to normal weight. Underweight was associated with more functional limitations compared to normal weight.	Expected association
Wardle et al., 2006 (70)	Depression in adolescent obesity: cultural moderators of the association between obesity and depressive symptoms	<u>HABITS study:</u> N = 4,320, age 11, 40.3% girls. <u>North West England study:</u> N = 1824, age 14–15, 68.4% girls.	1999	England	BMI, measured: IOTF.	Emotional symptoms & depressive symptoms, child reported: SDQ. Depressive symptoms, child reported: CES-D.	No association between BMI and emotional symptoms (I84HABITS study Obese vs. Normal weight: No association (emotional symptoms). Northwest England study Obese vs. Normal weight: No association (depressive symptoms).	No association

Wallander et al., 2009 (71)	Weight Status, Quality of Life, and Self-concept in African American, Hispanic, and White Fifth-grade Children	N = 599 (M = 10.80 years), 54% girls.	2003	United States	BMI, measured: CDC.	HRQoL, child reported: PedsQL. Self-perception, child reported: SPP.	Higher BMI was associated with lower psychosocial QOL and global self-concept.	Expected association
Bjørnelv et al., 2011 (72)	Psychological factors and weight problems in adolescents. The role of eating problems, emotional problems, and personality traits: the Young-HUNT study	N = 8,090, age 13-18 (M = 15.8), 50.3 % girls.	1995 - 1997	Norway	BMI, measured: Cole et al. (2000 & 2007). Dependent.	Self-esteem, child reported: RSES. Emotional symptoms, child reported: SCL-5. Independent.	Overweight and obesity was associated with lower self-esteem compared to normal weight. Among girls overweight was further associated to higher scores of emotional symptoms. Also, underweight was associated with higher self-esteem among girls.	Expected association and unexpected association
Wake et al., 2013 (73)	Morbidity patterns among the underweight, overweight and obese between 2 and 18 years: population-based cross-sectional analyses	<u>Toddlers:</u> N = 4,522, age 2-3 (M = 2.8), 49% girls. <u>Preschoolers:</u> N = 4,934, age 4-5 (M = 4.7), 49.2% girls. <u>Early primary:</u> N = 4,423, age 6-7 (M = 6.8), 49% girls. <u>Late primary:</u> N = 1,540, age 8-12 (M = 10.8), 49.9% female. <u>Adolescents:</u> N = 920, age 13-18 (M =15.9), 49.3% girls.	2000 - 2006	Australia	BMI, measured. Cole et al. (2000 & 2007). Independent.	HRQoL, parent reported: PedsQL. Mental health problems, parent reported (4-7 years): SDQ. Global health, parent reported: CHQ. Special health care needs, parent reported: CSHCN. Dependent.	BMI status was significantly associated with various health outcomes across all age groups. In toddlers, differences were seen in global health. Among preschoolers, BMI was linked to psychosocial health, mental health, global health, and special health-care needs. For children in early primary school, significant differences appeared in physical, psychosocial, mental, and global health. In late primary school, associations were found with physical and global health. Among adolescents, BMI status was related to physical, psychosocial, mental, and global health.	Expected association
Wallander et al., 2013 (74)	Is obesity associated with reduced health-related quality of life in Latino, black and white children in the community?	N = 4,824 (M = 11.12 years), 49% girls.	Not available	United States	BMI measured: CDC. Independent.	HRQoL, child reported: PedsQL. Self-perception, child reported: SPP. Dependent.	Overweight was associated with lower emotional HRQoL and global self-worth compared to normal weight. Obesity and extremely obesity was associated with lower total HRQoL, psychosocial HRQoL, social HRQoL and global self-worth compared to normal weight. Extremely obesity was further associated with physical HRQoL. Underweight was associated with	Expected association

							lower social HRQoL compared to normal weight.	
Lycett et al., 2018 (75)	Associations of mental health with cardiovascular risk phenotypes and adiposity in adolescence: A cross-sectional community-based study	N = 364, age 7-18 (M = 14.7), 54% girls.	2014	Australia	Waist circumference, bodyfat % & BMI, measured: IOTF. Dependent.	Physiological strengths and difficulties, child and parent reported: SDQ. Psychosocial health & general well-being, child and parent reported: PedsQL. Independent.	Higher BMI, body fat % & waist was associated with higher scores of emotional/behavioural problems and lower scores of psychosocial health and general well-being.	Expected association
Baile et al., 2020 (76)	The Relationship between Weight Status, Health-Related Quality of Life, and Life Satisfaction in a Sample of Spanish Adolescents	N = 1,197 (M = 14.4), 50.3% girls.	Not available	Spain	BMI, self-reported: Orbegozo foundation tables.	HRQoL, child reported: KIDSCREEN-10. Life satisfaction, child-reported: Cantril Scale.	Lower scores of HRQoL and life satisfaction among children with obesity vs. Normal weight.	Expected association
Alan et al., 2022 (77)	Mindful Eating, Body Weight, and Psycological Well-being in Adolescence	N = 401, age 14-18 (M = 15.9), 59.10% girls.	2019	Turkey	BMI, measured: CDC.	Self-esteem: RSES. Coping: WCI. Child reported.	Lowers scores in self-confident among children with obesity compared to normal weight. Lower scores of helpless and RSES among children with overweight compared to normal weight.	Expected association

*Abbreviations: BMI = *Body Mass Index*; CDC = *Centers for Disease Control and Prevention*; CHU9D = *The Child Health Utility 9D*; CHQ-PF50 = *Child Health Questionnaire - Parent Form 50*; CRS = *The Conner's Rating Scale*; DEXA-scan = *Dual Energy X-ray Absorptiometry Scan*; DI = *Dominic Interactive;* EAT 2010 Survey = *Eating Assessment Tool* - 235-item assessing a range of factors of potential relevance to weight status and weight-related behaviors among adolescent; EQ-5D = *European Quality of life – 5 Dimensions*; HRQoL = *Health Related Quality of Life*; HSS = *The Hare Self-Esteem* Scale; IOTF = *International Obesity Task Force;* IWQOL-kids = *The Impact of Weight on Quality of Life – Kids*; KIDSCREEN = 10/27/52 items health-related quality of life questionnaire; KINDL = German Questionnaire on HRQoL; LAWSEQ = *The Lawrence's Self-Esteem Questionnaire*; PANAS-C = *The Positive Affect and Negative Affect Schedule– Child Form*; PedsQL = *Pediatric Quality of Live Inventory*; QoL = *Quality of Life*; PHCSCS(-2) = *Piers Harris Children's Self-Concept Scale (Second Edition)*; RSE = *Rosenberg Self Esteem*; SDQ = *Strengths and Difficulties Questionnaire*; SF-12/36 = *Short Form 12/36;* SPPA/SPPC = *The Harter Self-Perception Profile for Adolescents/Children*; SRS = *Social Rating Scale - adapted from the Social Skill Rating System*; PSS = *Perceived Stress Scale*; SSRS = *Social Skills Rating System;* WHO = *World Health Organization*; WHtR = *Waist-to-height ratio.*

**Included studies have different study designs but are included based on the relevant analysis.

References

1. Wake M, Salmon L, Waters E, Wright M, Hesketh K. Parent-reported health status of overweight and obese Australian primary school children: a cross-sectional population survey. Int J Obes. 2002;26(5):717–24. doi: 10.1038/sj.ijo.0801974.

2. Friedlander SL, Larkin EK, Rosen CL, Palermo TM, Redline S. Decreased Quality of Life Associated With Obesity in School-aged Children. Arch Pediatr Adolesc Med. 2003;157(12):1206-11. doi: 10.1001/archpedi.157.12.1206.

3. Pinhas-Hamiel O, Singer S, Pilpel N, Fradkin A, Modan D, Reichman B. Health-related quality of life among children and adolescents: associations with obesity. Int J Obes. 2006;30(2):267–72. doi: 10.1038/sj.ijo.0803107

4. De Beer M, Hofsteenge GH, Koot HM, Hirasing RA, Delemarre-Van De Waal HA, Gemke RJBJ. Health-related-quality-of-life in obese adolescents is decreased and inversely related to BMI. Acta Paediatr. 2007;96(5):710–4. doi: 10.1111/j.1651-2227.2007.00243.x

5. Vissers D, Devoogdt N, Gebruers N, Mertens I, Truijen S, Van Gaal L. Overweight in Adolescents: Differences per Type of Education. Does One Size Fit All? J Nutr Educ Behav. 2008;40(2):65–71. doi: 10.1016/j.jneb.2007.06.010.

6. Boyle SE, Jones GL, Walters SJ. Physical activity, quality of life, weight status and diet in adolescents. Qual Life Res. 2010;19(7):943–54. doi: 10.1007/s11136-010-9659-8.

7. Haraldstad K, Christophersen K, Eide H, Nativg GK, Helseth S. Predictors of health-related quality of life in a sample of children and adolescents: a school survey. J Clin Nurs. 2011;20(21–22):3048–56. doi: 10.1111/j.1365-2702.2010.03693.x

8. Keating CL, Moodie ML, Swinburn BA. The health-related quality of life of overweight and obese adolescents--a study measuring body mass index and adolescent-reported perceptions. Int J Pediatr Obes. 2011;6(5–6):434–41. doi: 10.3109/17477166.2011.590197

9. Van Grieken A, Veldhuis L, Renders CM, Landgraf JM, Hirasing RA, Raat H. Impaired parent-reported health-related quality of life of underweight and obese children at elementary school entry. Qual Life Res. 2013;22(4):917–28. doi: 10.1007/s11136-012-0211-x.

10. Kesztyüs D, Wirt T, Kobel S, et al. Is central obesity associated with poorer health and health-related quality of life in primary school children? Cross-sectional results from the Baden-Württemberg Study. BMC Public Health. 2013;13:260. doi: 10.1186/1471-2458-13-260.

11. Morales PF, Sánchez-López M, Moya-Martínez P, et al. Health-related quality of life, obesity, and fitness in schoolchildren: the Cuenca study. Qual Life Res. 2013;22(7):1515–23. doi: 10.1007/s11136-012-0282-8.

12. Petracci E, Cavrini G. The effect of weight status, lifestyle, and body image perception on health-related quality of life in children: a quantile approach. Qual Life Res. 2013;22(9):2607–15. doi: 10.1007/s11136-013-0358-0.

13. Chen G, Ratcliffe J, Olds T, Magarey A, Jones M, Leslie E. BMI, Health Behaviors, and Quality of Life in Children and Adolescents: A School-Based Study. Pediatrics. 2014;133(4):e868–74. doi: 10.1542/peds.2013-0622.

14. Cui W, Zack MM, Wethington H. Health-related quality of life and body mass index among US adolescents. Qual Life Res. 2014;23(7):2139–50. doi: 10.1007/s11136-014-0646-3.

15. Gandhi PK, Revicki DA, Huang IC. Adolescent body weight and health-related quality of life rated by adolescents and parents: The issue of measurement bias Energy balance-related behaviors. BMC Public Health. 2015;15(1). doi: 10.1186/s12889-015-2533-4.

16. Helseth S, Haraldstad K, Christophersen KA. A cross-sectional study of Health Related Quality of Life and body mass index in a Norwegian school sample (8-18 years): A comparison of child and parent perspectives. Health Qual Life Outcomes. 2015;13(1). doi: 10.1186/s12955-015-0239-z.

17. Parkinson KN, Adamson AJ, Basterfield L, Reilly JK, Le Couteur A, Reilly JJ. Influence of adiposity on health-related quality of life in the Gateshead Millennium Study cohort: longitudinal study at 12 years. Arch Dis Child. 2015;100(8):779–83. doi: 10.1136/archdischild-2014-307498.

18. Meyer M, Oberhoffer R, Hock J, Giegerich T, Müller J. Health-related quality of life in children and adolescents: Current normative data, determinants and reliability on proxy-report. J Paediatrics Child Health. 2016;52(6):628–31. doi: 10.1111/jpc.13166.

19. Mitchell TB, Steele RG. The Effect of Body Mass Index, Negative Affect, and Disordered Eating on Health-Related Quality of Life in Preadolescent Youth. J Pediatr Psychol. 2016;41(7):768–76. doi: 10.1093/jpepsy/jsv163.

20. Andersen JR, Natvig GK, Aadland E, et al. Associations between health-related quality of life, cardiorespiratory fitness, muscle strength, physical activity and waist circumference in 10-year-old children: the ASK study. Qual Life Res. 2017;26(12):3421–8. doi: 10.1007/s11136-017-1634-1.

21. Muros JJ, Salvador Pérez F, Zurita Ortega F, Gámez Sánchez VM, Knox E. The association between healthy lifestyle behaviors and health-related quality of life among adolescents. J Pediatr (Rio J). 2017;93(4):406–12. doi: 10.1016/j.jped.2016.10.005.

22. Tsiros MD, Samaras MG, Coates AM, Olds T. Use-of-time and health-related quality of life in 10- to 13-year-old children: not all screen time or physical activity minutes are the same. Qual Life Res. 2017;26(11):3119–29. doi: 10.1007/s11136-017-1639-9.

23. Halasi S, Lepeš J, Đorđić V, et al. Relationship between obesity and health-related quality of life in children aged 7–8 years. Health Qual Life Outcomes. 2018;16(1):149. doi: 10.1186/s12955-018-0974-z.

24. Perez-Sousa MA, Olivares PR, Escobar-Alvarez JA, Parraça JA, Gusi N. Fitness as mediator between weight status and dimensions of health-related quality of life. Health Qual Life Outcomes. 2018;16(1):155. doi: 10.1186/s12955-018-0981-0.

25. Hoare E, Crooks N, Hayward J, Allender S, Strugnell C. Associations between combined overweight and obesity, lifestyle behavioural risk and quality of life among Australian regional school children: baseline findings of the Goulburn Valley health behaviours monitoring study. Health Qual Life Outcomes. 2019;17(1):16. doi: 10.1186/s12955-019-1086-0.

26. Bottolfs M, Støa EM, Reinboth MS, et al. Resilience and lifestyle-related factors as predictors for health-related quality of life among early adolescents: a cross-sectional study. J Int Med Res. 2020;48(2):030006052090365. doi: 10.1177/0300060520903656.

27. Killedar A, Lung T, Petrou S, Teixeira-Pinto A, Tan EJ, Hayes A. Weight status and health-related quality of life during childhood and adolescence: effects of age and socioeconomic position. Int J Obes (Lond). 2020;44(3):637–45. doi: 10.1038/s41366-020-0529-3.

28. Çam HH, Üstüner Top F. Overweight, obesity, weight-related behaviors, and health-related quality of life among high-school students in Turkey. Eat Weight Disord. 2020;25(5):1295–302. doi: 10.1007/s40519-019-00762-0.

29. Meixner L, Cohrdes C, Schienkiewitz A, Mensink GBM. Health-related quality of life in children and adolescents with overweight and obesity: results from the German KIGGS survey. BMC Public Health. 2020;20(1):1722. doi: 10.1186/s12889-020-09834-8.

30. Thumann BF, Börnhorst C, Ahrens W, et al. Cross-Sectional and Longitudinal Associations Between Psychosocial Well-Being and Cardiometabolic Markers in European Children and Adolescents. Psychosom Med. 2020;82(8):764–73. doi: 10.1097/PSY.0000000000000845.

31. Mastorci F, Piaggi P, Doveri C, et al. Relationship between Weight Status and Health-Related Quality of Life in a Sample of Early Adolescents from Central and Northern Italy: A Cross-Sectional Study of the AVATAR Project Participants. IJERPH. 2021;18(16):8782. doi: 10.3390/ijerph18168782.

32. Hassan H, Paulis WD, Bindels PJE, Koes BW, Van Middelkoop M. Somatic complaints as a mediator in the association between body mass index and quality of life in children and adolescents. BMC Fam Pract. 2021;22(1):214. doi: 10.1186/s12875-021-01562-1.

33. Masini A, Gori D, Marini S, et al. The Determinants of Health-Related Quality of Life in a Sample of Primary School Children: A Cross-Sectional Analysis. IJERPH. 2021;18(6):3251. doi: 10.3390/ijerph18063251.

34. Papadaki S, Carayanni V, Notara V, Chaniotis D. Anthropometric, Lifestyle Characteristics, Adherence to the Mediterranean Diet, and COVID-19 Have a High Impact on the Greek Adolescents' Health-Related Quality of Life. Foods. 2022;11(18):2726. doi: 10.3390/foods11182726.

35. Alanne S, Koivuniemi E, Löyttyniemi E, Laitinen K. Elementary School-Aged Children's and Parents' Report of Health-Related Quality of Life and Relationships with Lifestyle Measures: A Cross-Sectional Study. Nutrients. 2023;15(5):1264. doi: 10.3390/nu15051264.

36. Ródenas-Munar M, Monserrat-Mesquida M, Gómez SF, et al. Perceived Quality of Life Is Related to a Healthy Lifestyle and Related Outcomes in Spanish Children and Adolescents: The Physical Activity, Sedentarism, and Obesity in Spanish Study. Nutrients. 2023;15(24):5125. doi: 10.3390/nu15245125.

37. Rodrigues D, Machado-Rodrigues AM, Gama A, Silva MG, Nogueira H, Padez C. Body size, form, composition, and a healthy lifestyle associates with health-related quality of life among Portuguese children. Am J Hum Biol. 2023;35(8):e23902. doi: 10.1002/ajhb.23902.

38. Phillips R, Hill A. Fat, plain, but not friendless: self-esteem and peer acceptance of obese pre-adolescent girls. Int J Obes. 1998;22(4):287–93. doi: 10.1038/sj.ijo.0800582.

39. Pesa JA, Syre TR, Jones E. Psychosocial differences associated with body weight among female adolescents: the importance of body image. J Adolesc Health. 2000;26(5):330–7. doi: 10.1016/s1054-139x(99)00118-4.

40. Gordon-Larsen P. Obesity-related knowledge, attitudes, and behaviors in obese and non-obese urban Philadelphia female adolescents. Obes Res. 2001;9(2):112–8. doi: 10.1038/oby.2001.14.

41. Mirza NM, DavisM.S. D, Yanovski JA. Body dissatisfaction, self-esteem, and overweight among inner-city Hispanic children and adolescents. J Adolesc Health. marts 2005;36(3):267.e16-267.e20. doi: 10.1016/j.jadohealth.2004.02.033.

42. Allen KL, Byrne SM, Blair EM, Davis EA. Why do some overweight children experience psychological problems? The role of weight and shape concern. Int J Pediatr Obes. 2006;1(4):239–47. doi: 10.1080/17477160600913552.

43. Kristjánsson AL, Sigfúsdóttir ID, Allegrante JP. Health behavior and academic achievement among adolescents: the relative contribution of dietary habits, physical activity, body mass index, and self-esteem. Health Educ Behav. 2010;37(1):51–64. doi: 10.1177/1090198107313481.

44. McCullough N, Muldoon O, Dempster M. Self-perception in overweight and obese children: a cross-sectional study. Child Care Health Dev. 2009;35(3):357–64. doi: 10.1111/j.1365-2214.2008.00924.x.

45. Danielsen YS, Stormark KM, Nordhus IH, et al. Factors Associated with Low Self-Esteem in Children with Overweight. Obes Facts. 2012;5(5):722–33. doi: 10.1159/000338333.

46. Willows ND, Ridley D, Raine KD, Maximova K. High adiposity is associated cross-sectionally with low self-concept and body size dissatisfaction among indigenous Cree schoolchildren in Canada. BMC Pediatr. 2013;13(1):118. doi: 10.1186/1471-2431-13-118.

47. Wong WW, Mikhail C, Ortiz CL, et al. Body weight has no impact on self-esteem of minority children living in inner city, low-income neighborhoods: a cross-sectional study. BMC Pediatr. 2014;14(1):19. doi: 10.1186/1471-2431-14-19.

48. Altıntaş A, Aşçı FH, Kin-İşler A, et al. The role of physical activity, body mass index and maturity status in body-related perceptions and self-esteem of adolescents. Ann Hum Biol. 2014;41(5):395–402. doi: 10.3109/03014460.2013.857721.

49. Bauer KW, Marcus MD, Larson N, Neumark-Sztainer D. Socioenvironmental, Personal, and Behavioral Correlates of Severe Obesity among an Ethnically/Racially Diverse Sample of US Adolescents. Child Obes. 2017;13(6):470–8. doi: 10.1089/chi.2017.0067.

50. Guillamón AR, García Cantó E, Carrillo López PJ. Weight status, physical activity and self-concept in primary school children. jhse. 2019;14(3), 515-526. doi: 10.14198/jhse.2019.143.03

51. Vassiloudis I, Costarelli V. Excess body weight and abdominal obesity in relation to selected psychosocial characteristics in primary school children. NFS. 2019;50(4):739–50. doi: 10.1108/NFS-06-2019-0184

52. Blanco M, Solano S, Alcántara AI, Parks M, Román FJ, Sepúlveda AR. Psychological well-being and weight-related teasing in childhood obesity: a case–control study. Eat Weight Disord. 2020;25(3):751–9. doi: 10.1007/s40519-019-00683-y

53. Altan SS, Bektas M. The effects of parental feeding styles, children's self-efficacy and social anxiety on adolescent obesity. Rev Nutr. 2017;30(6):759–69. doi: 10.1590/1678-98652017000600008

54. Sawyer MG, Miller-Lewis L, Guy S, Wake M, Canterford L, Carlin JB. Is There a Relationship Between Overweight and Obesity and Mental Health Problems in 4- to 5-Year-Old Australian Children? Ambul Pediatr. 2006;6(6):306–11. doi: 10.1016/j.ambp.2006.08.005.

55. Pitrou I, Shojaei T, Wazana A, Gilbert F, Kovess-Masféty V. Child Overweight, Associated Psychopathology, and Social Functioning: A French School-based Survey in 6- to 11-year-old Children. Obesity. 2010;18(4):809–17. doi: 10.1038/oby.2009.278.

56. Pastor PN, Reuben CA. Emotional/behavioral difficulties and adolescent obesity: effect of sex and Hispanic origin/race. Int J Pediatr Obes. 2011;6(5–6):462–6. doi: 10.3109/17477166.2011.590207.

57. Lu E, Dayalu R, Diop H, Harvey EM, Manning SE, Uzogara SG. Weight and Mental Health Status in Massachusetts, National Survey of Children's Health, 2007. Matern Child Health J. 2012;16(S2):278–86. doi: 10.1007/s10995-012-1145-1.

58. Halfon N, Larson K, Slusser W. Associations Between Obesity and Comorbid Mental Health, Developmental, and Physical Health Conditions in a Nationally Representative Sample of US Children Aged 10 to 17. Acad Pediatr. 2013;13(1):6–13. doi: 10.1016/j.acap.2012.10.007.

59. Kozieł S, Chakraborty R, Bose K. Relationship between temperament and fatness in 11-year-old children and 17-year-old adolescents from Wrocław, Poland. Homo. 2017;68(6):479–86. doi: 10.1016/j.jchb.2017.10.002.

60. Noonan RJ, Fairclough SJ. Cross-sectional associations between body mass index and social–emotional wellbeing among differentially active children. Eur J Public Health. 2019;29(2):303–7. doi: 10.1093/eurpub/cky208.

61. Åvitsland A, Leibinger E, Haugen T, et al. The association between physical fitness and mental health in Norwegian adolescents. BMC Public Health. 2020;20(1):776. doi: 10.1186/s12889-020-08936-7.

62. Bjertnaes AA, Fossum IN, Oma I, Bakken KS, Arne T, Holten-Andersen MN. A Cross-Sectional Study of the Relationship Between Mental Health Problems and Overweight and Obesity in Adolescents. Front Public Health. 2020;8:334. doi: 10.3389/fpubh.2020.00334.

63. Lundgren O, Henriksson P, Delisle Nyström C, Silfvernagel K, Löf M. Hyperactivity is associated with higher fat-free mass and physical activity in Swedish preschoolers: A cross-sectional study. Acta Paediatr. 2021;110(4):1273–80. doi: 10.1111/apa.15608.

64. López-Gil JF, Cavero-Redondo I, Jiménez-López E, Bizzozero-Peroni B, Saz-Lara A, Mesas AE. Role of Diet Quality in the Association Between Excess Weight and Psychosocial Problems in a Large Sample of Children in Spain. JAMA Netw Open. 2022;5(4):e229574. doi: 10.1001/jamanetworkopen.2022.9574.

65. Chen S, Zhang H, Gao M, et al. Dose-Dependent Association Between Body Mass Index and Mental Health and Changes Over Time. JAMA psychiatry. 2024;81(8):797–806. doi: 10.1001/jamapsychiatry.2024.0921.

66. Marques A, De Matos MG. Trends in prevalence of overweight and obesity: are Portuguese adolescents still increasing weight? Int J Public Health. 2016;61(1):49–56. doi: 10.1007/s00038-015-0758-8.

67. Chai L, Xue J. Weight, Weight Perceptions, and Health and Well-Being Among Canadian Adolescents: Evidence From the 2017-2018 Canadian Community Health Survey. Am J Health Promot. 2022;36(1):55–63. doi: 10.1177/08901171211031064.

68. Trombetta CM, Marconi D, Lipari D, et al. There is a correlation between nutritional status, Self-Rated Health and Life Satisfaction? Evidence from 2018 Health Behaviour in School-aged Children cross sectional study in a sample of Italian adolescents living in Tuscany Region. J Prev Med Hyg. 2024;65(1):E43–9. doi: 10.15167/2421-4248/jpmh2024.65.1.3178.

69. Swallen KC, Reither EN, Haas SA, Meier AM. Overweight, Obesity, and Health-Related Quality of Life Among Adolescents: The National Longitudinal Study of Adolescent Health. Pediatrics. 2005;115(2):340–7. doi: 10.1542/peds.2004-0678.

70. Wardle J, Williamson S, Johnson F, Edwards C. Depression in adolescent obesity: cultural moderators of the association between obesity and depressive symptoms. Int J Obes (Lond). 2006;30(4):634–43. doi: 10.1038/sj.ijo.0803142.

71. Wallander JL, Taylor WC, Grunbaum JA, et al. Weight status, quality of life, and self-concept in African American, Hispanic, and white fifth-grade children. Obesity (Silver Spring). 2009;17(7):1363–8. doi: 10.1038/oby.2008.668.

72. Bjornelv S, Nordahl HM, Holmen TL. Psychological factors and weight problems in adolescents. The role of eating problems, emotional problems, and personality traits: the Young-HUNT study. Soc Psychiatry Psychiatr Epidemiol. 2011;46(5):353–62. doi: 10.1007/s00127-010-0197-z.

73. Wake M, Clifford SA, Patton GC, et al. Morbidity patterns among the underweight, overweight and obese between 2 and 18 years: population-based cross-sectional analyses. Int J Obes (Lond). 2013;37(1):86–93. doi: 10.1038/ijo.2012.86.

74. Wallander JL, Kerbawy S, Toomey S, et al. Is obesity associated with reduced health-related quality of life in Latino, black and white children in the community? Int J Obes (Lond). 2013;37(7):920–5. doi: 10.1038/ijo.2013.31

75. Lycett K, McNamara C, Mensah FK, et al. Associations of mental health with cardiovascular risk phenotypes and adiposity in adolescence: A cross-sectional community-based study. J Paediatr Child Health. 2018;54(6):677–84. doi: 10.1111/jpc.13943.

76. Baile JI, Guevara RM, González-Calderón MJ, Urchaga JD. The Relationship between Weight Status, Health-Related Quality of Life, and Life Satisfaction in a Sample of Spanish Adolescents. IJERPH. 2020;17(9):3106. doi: 10.3390/ijerph17093106.

77. Alan BE, Akdemir D, Cetin FC, Karahan S. Mindful Eating, Body Weight, and Psychological Well-Being in Adolescence. 2022;18(4):246-253. doi: 10.1089/chi.2021.0121.
